# Supplementary material for: CREG1 restricts ALV-J replication via the mitochondrial dysfunction–driven activation of innate immunity and apoptosis
Source: Front Immunol. 2026 Jan 21;16:1760120. doi: 10.3389/fimmu.2025.1760120 (PMC12867876; doi:10.3389/fimmu.2025.1760120)
Supplement: Supplementary file 1 [file SupplementaryFile1.docx]

**Supplementary Figures**


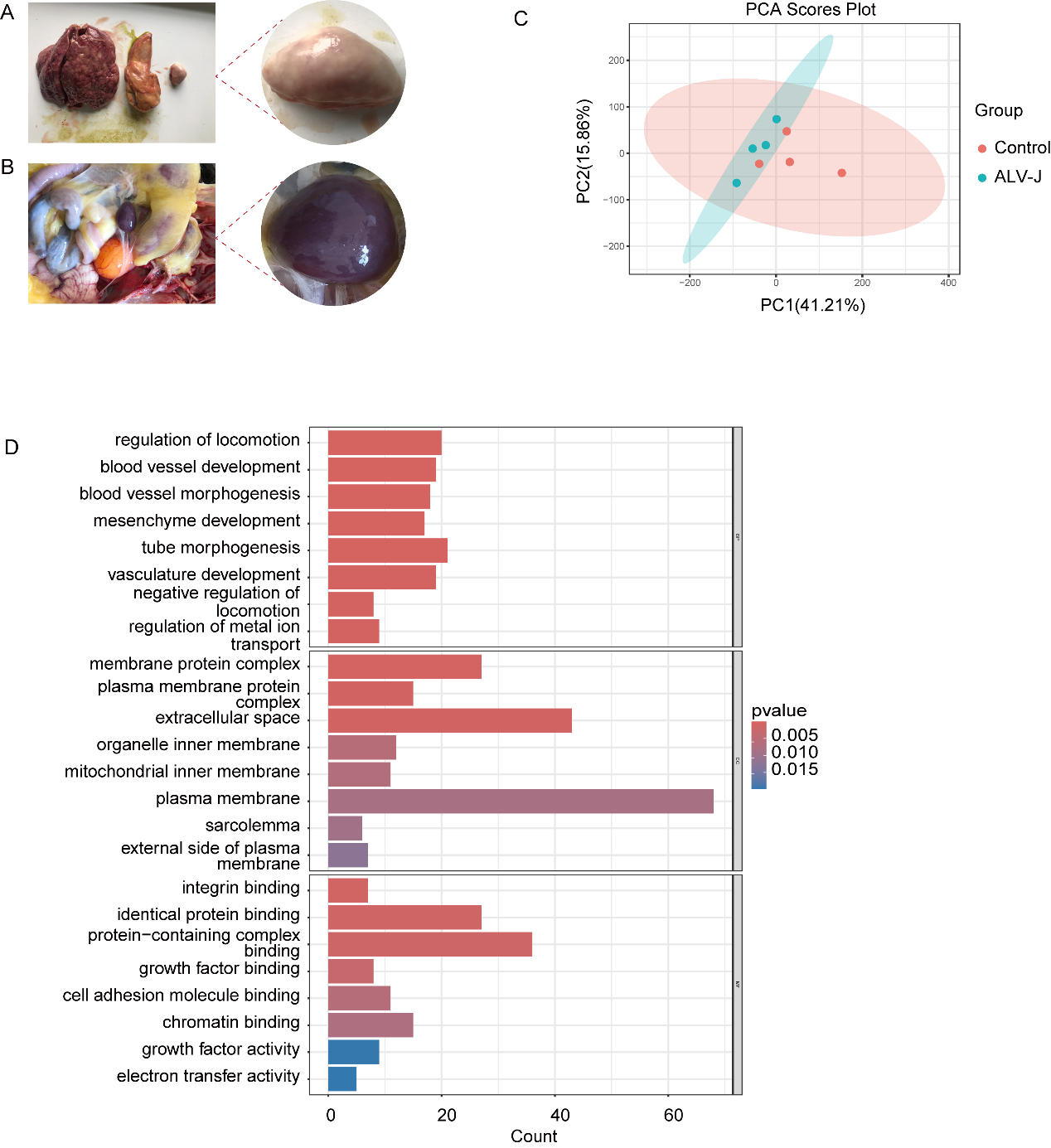


**Figure S1. Transcriptome sequencing was performed on spleen tissues collected from ALV-J-infected chickens.**

**(A).** Spleen tissues from chickens with ALV-J-infected.

**(B).** Spleen tissues from healthy control chickens.

**(C).** Principal component analysis (PCA) results between the experimental and control groups. n = 4.

**(D).** Gene ontology (GO) enrichment analysis of differentially expressed genes. The experimental and control groups each had four replicates. Differentially expressed genes were identified based on the criteria of |log2FC| ≥ 1 and p-value ≤ 0.01.

**
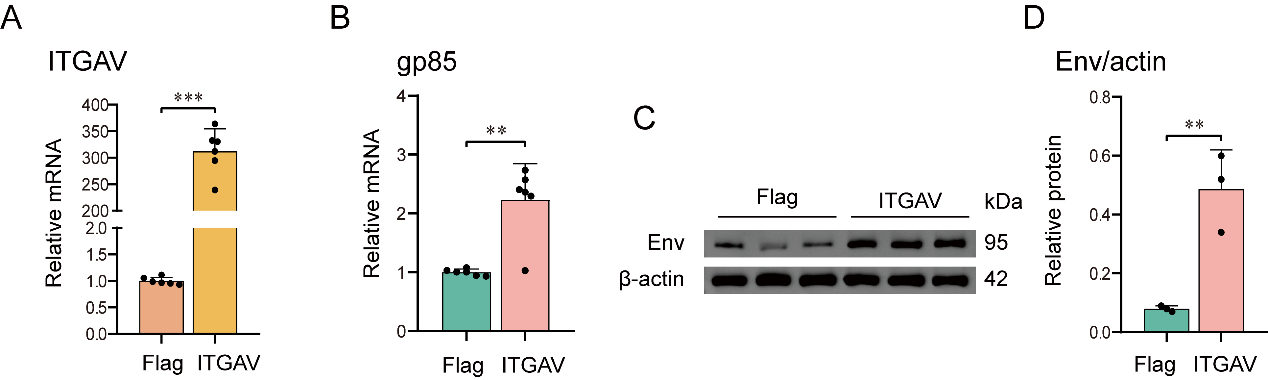
**

**Figure S2. Detection of ALV-J replication upon ITGAV overexpression.**

**(A).** Expression efficiency was assessed by reverse transcription quantitative polymerase chain rection (RT-qPCR) 24 hours after transfection of Flag-ITGAV into DF-1 cells. n = 6.

**(B).** After transfecting DF-1 cells with Flag-ITGAV or the control group, followed by ALV-J infection, the expression level of the viral protein gp85 was measured by RT-qPCR 48 hours post-infection (hpi). n = 6.

**(C).** After transfecting DF-1 cells with Flag-ITGAV or the control group, followed by ALV-J infection, the expression level of the viral envelope protein env and β-actin were measured by western blotting 48 hpi. n = 3.

**(D).** Relative expression bar graph of env protein (Env/actin).

For **A**, **B** and **D**, the values are shown as the mean ± SD. *p < 0.05, **p < 0.01, ***p < 0.001 by unpaired Student’s t test.

**
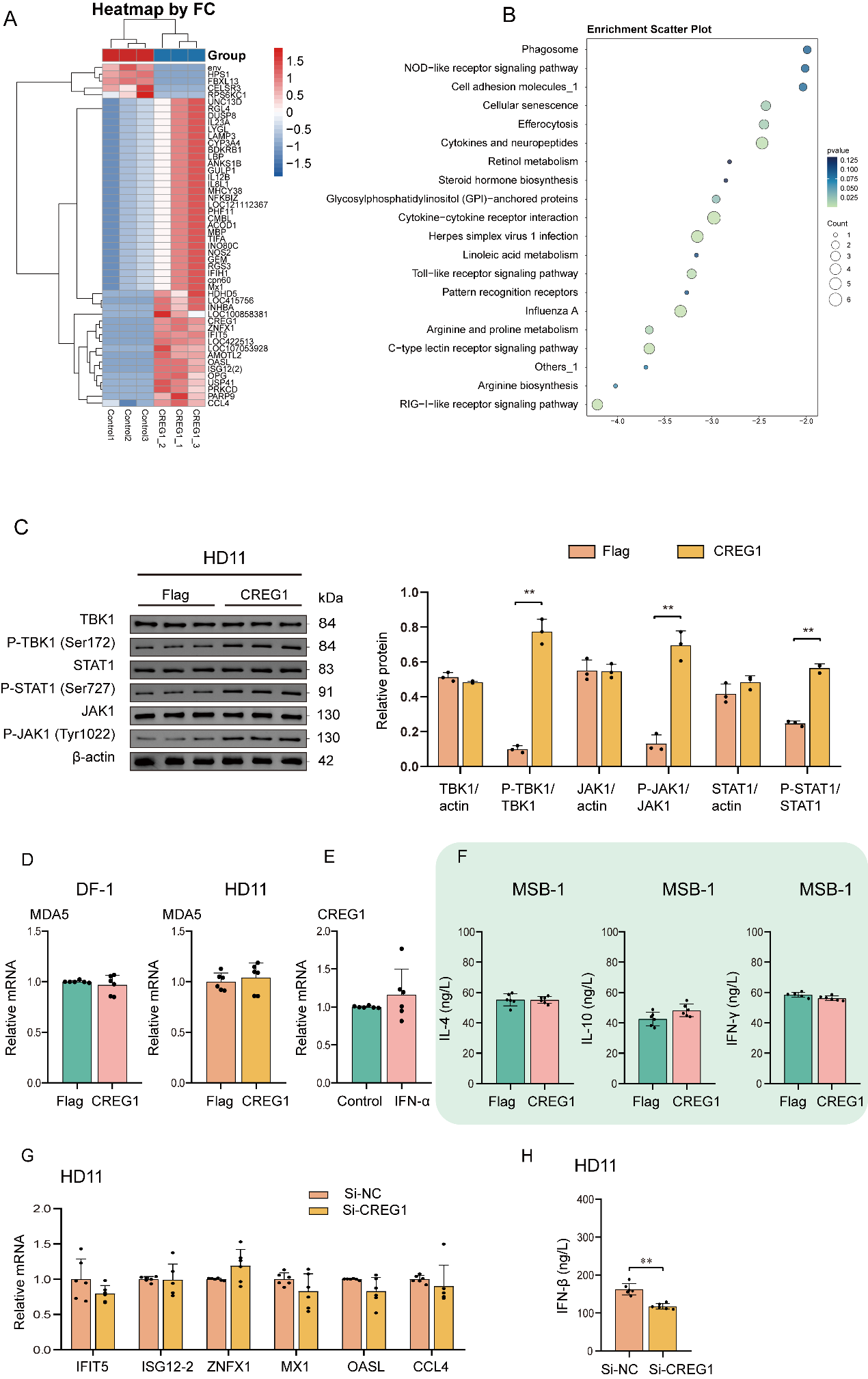
**

**Figure S3. *CREG1* functions as a trigger for innate immune defense.**

**(A-B).** Overexpression of *CREG1* or control in DF-1 cells infected with ALV-J, with proteomic results at 48 hpi. The heatmap (**A**) shows the expression of differential proteins based on fold change (FC), the KEGG pathway enrichment map of differential proteins (**B**). n = 3.

**(C).** *CREG1* or control overexpression in HD11 followed by ALV-J infection, with western blot analysis at 48 hpi to assess the activation of the I-IFN signaling pathway, including TBK1, P-TBK1 (Ser172), STAT1, P-STAT1 (Ser727), JAK, P-JAK (Tyr1022), and actin. The left panel shows the immunoblotting results, while the right panel presents the densitometric analysis of relative protein expression. n = 3.

**(D).** ALV-J was infected in *CREG1*-overexpressing DF-1 or HD11 cells, and the expression of *MDA5* was detected by RT-qPCR at 48 hpi. n = 6.

**(E).** HD11 cells were stimulated with IFN-α (500 IU/mL for 12h), and the expression of CREG1 was detected by RT-qPCR at 48 hpi. n = 6.

**(F).** ALV-J was infected in *CREG1*-overexpressing MSB-1 cells, and the levels of IL-4 (left), IL-10 (middle), and IFN-γ (right) in the cell supernatant were measured by Enzyme-Linked Immunosorbent Assay (ELISA) at 48 hpi. n = 6.

**(G).** After *CREG1* was silenced by siRNA in HD11 cells, ALV-J was infected, and the expression levels of interferon-stimulated genes (ISGs) were measured by RT-qPCR at 48 hpi. n = 6.

**(H).** After *CREG1* was silenced by siRNA in HD11 cells, ALV-J was infected, and the level of IFN-β in the cell supernatant was measured by ELISA at 48 hpi.

For **D-H**, the values are shown as the mean ± SD. n = 6. *p < 0.05, **p < 0.01, ***p < 0.001 by unpaired Student’s t test.


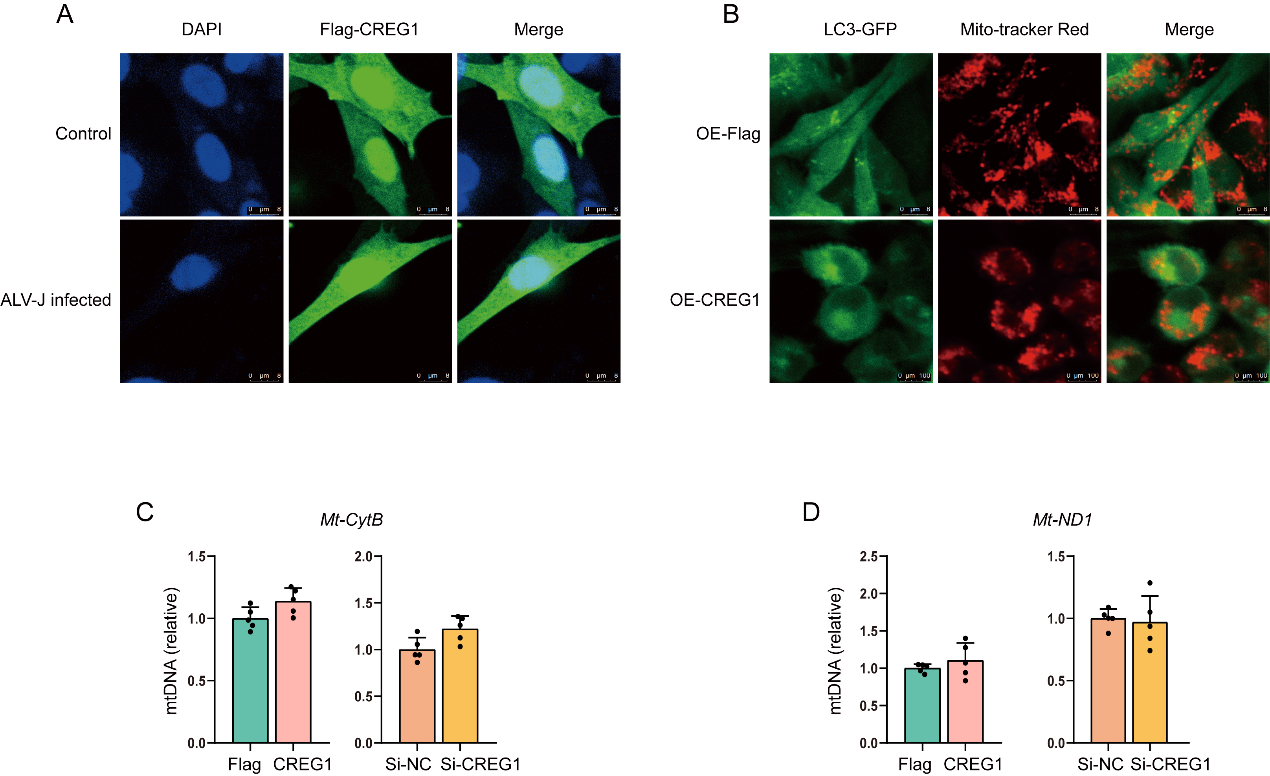


**Figure S4. *CREG1* induces mitophagy.**

**(A).** Immunoblotting of nuclear and cytoplasmic fractions was performed using an anti-FLAG antibody, followed by a FITC-conjugated secondary antibody. Scale bars, 8 µm.

**(B).** DF-1 cells were transfected with Flag-*CREG1* or control plasmid, and after 6 hours, the medium was replaced with fresh medium followed by infection with lentivirus expressing GFP-LC3B. Twenty-four hours later, the cells were infected with ALV-J. At 24hpi, mitochondria were labeled with MitoTracker Deep Red, and the localization of GFP-LC3B was examined using confocal microscopy. Co-localization of GFP-LC3B with MitoTracker Deep Red was indicative of mitophagy. Scale bars, 8 µm.

**(C-D).** Total mtDNA levels in whole-cell lysates were determined in CREG1-overexpressing (**C**) and CREG1-knockdown cells (**D**) following ALV-J infection at 48 hpi. n=6.

For **C-D**, the values are shown as the mean ± SD. *p < 0.05, **p < 0.01, ***p < 0.001 by unpaired Student’s t test.

**
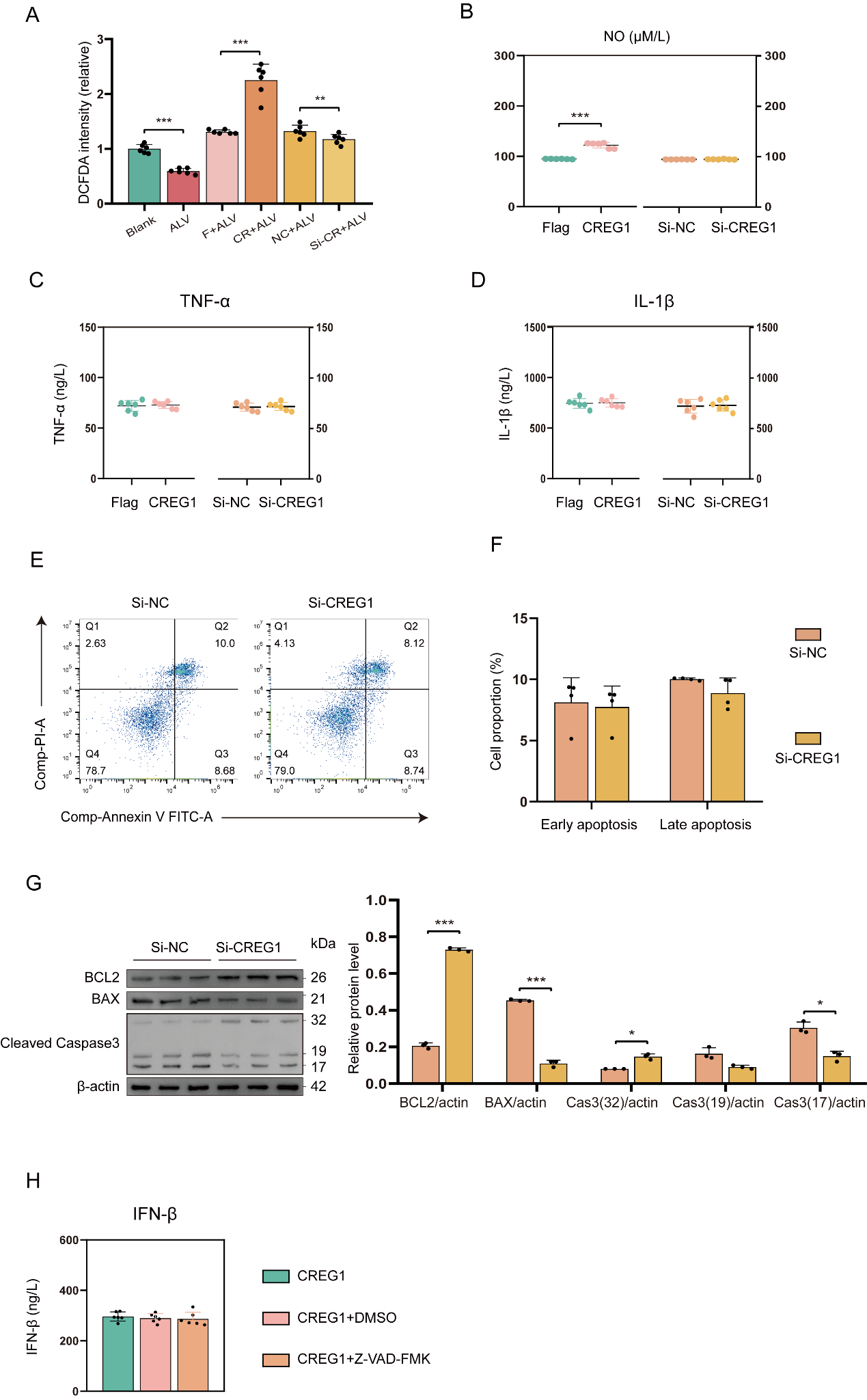
**

**Figure S5. Apoptosis induction by *CREG1* is critical for its suppression of viral replication.**

**(A-B).** DF-1 cells with *CREG1* overexpression or knockdown were infected with ALV-J, and at 48 hpi, cells were collected for DCFDA (**A**) and NaNO2 (**B**) fluorescence measurement using a microplate reader. n = 6.

**(C-D).** The levels of TNF-α (**C**) and IL-1β (**D**) in the supernatants of *CREG1*-overexpressing or knockdown cells infected with ALV-J were measured by ELISA at 48 hpi. n = 6.

**(E-G).** Cells transfected with siRNA targeting *CREG1* were infected with ALV-J. Apoptosis was evaluated by flow cytometry (**E-F**), and apoptosis-associated protein expression was detected by Western blot (**G**) at 48 hpi. n = 4 (**E**) or n = 3 (**G**).

**(H).** After 24 hours of *CREG1* overexpression, cells were pretreated with Z-VAD-FMK (50 μM) or DMSO for 1 hour prior to ALV-J infection. At 24 hpi, IFN-β levels in the culture supernatants were measured by ELISA. n = 6.

For **A**, **B**, **G**, the values are shown as the mean ± SD. *p < 0.05, **p < 0.01, ***p < 0.001 by unpaired Student’s t test.

**
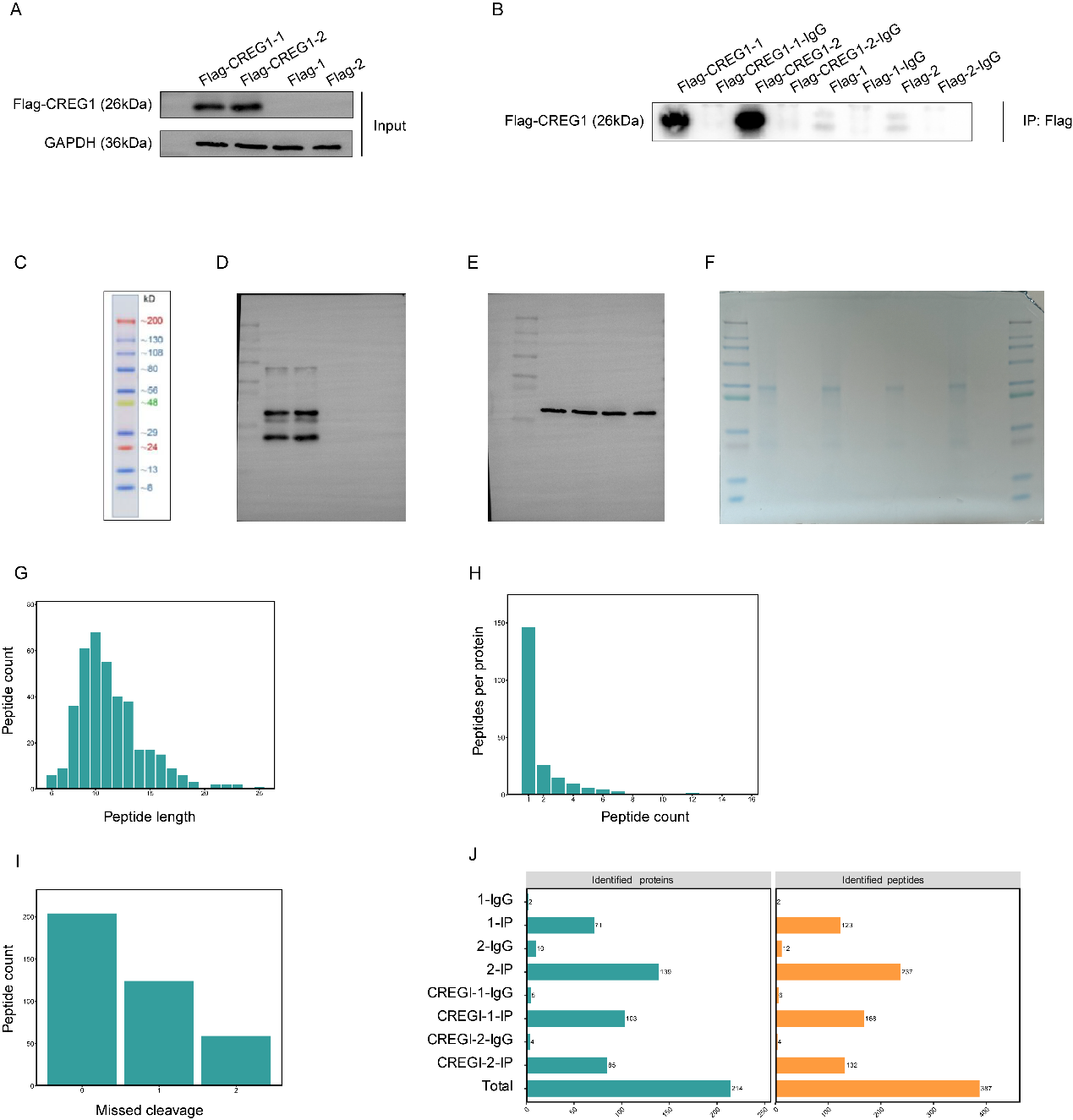
**

**Figure S6.** **DF-1 cells overexpressing Flag-tagged *CREG1* were infected with ALV-J, followed by mass spectrometry analysis.**

**(A-J).** DF-1 cells overexpressing Flag-*CREG1* were infected with ALV-J, and cells were harvested at 48 hpi for immunoprecipitation. (**A**) Immunoblot of input samples using anti-Flag antibody and GAPDH as a loading control. (**B**) Immunoblot of proteins enriched with anti-Flag antibody. (**C**) 200 kDa protein marker. (**D-E**) Full, uncropped immunoblot membranes. (**F**) Coomassie Brilliant Blue staining. (**G-J**) Results of peptide identification.

**
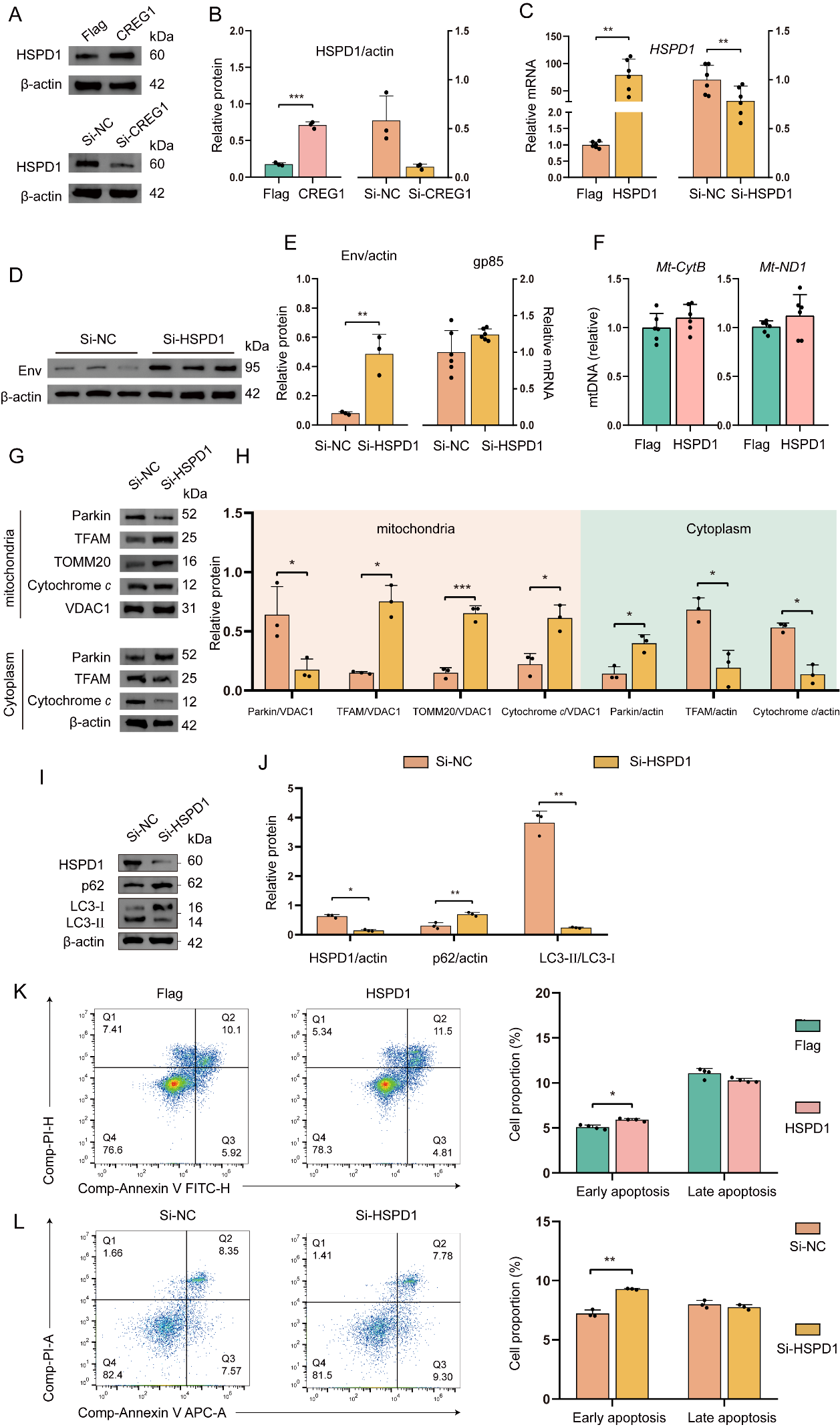
**

**Figure S7. *HSPD1* suppresses ALV-J replication and modulates mitochondrial function.**

**(A-B).** Western blot analysis of *HSPD1* expression following *CREG1* overexpression or knockdown. **(A)** Representative Western blot images. **(B)** Quantification of relative protein levels based on grayscale intensity. n = 3.

**(C).** RT-qPCR analysis of *HSPD1* expression 48 hours after transfection with HA-*HSPD1* or siRNA. n = 6.

**(D-E).** Following *HSPD1* knockdown, cells were infected with ALV-J, and viral expression levels were measured by Western blot or RT-qPCR at 48 hpi. n = 3/6.

**(F).** Total cellular mtDNA levels were measured in CREG1-overexpressing cells infected with ALV-J at 48 hpi. n=6.

**(G-H).** Western blot analysis of Parkin, TFAM, TOMM20, Cytochrome C, and VDAC1 in mitochondrial and cytosolic fractions of *HSPD1* knockdown cells at 48 hpi with ALV-J infection. (**G**) Representative Western blot images. (**H**) Quantification of relative protein levels based on grayscale intensity. n = 3.

**(I-J).** Western blot analysis of p62 and LC3 expression in total cell lysates of *HSPD1* knockdown cells at 48 hpi after ALV-J infection. (**I**) Representative Western blot images. (**J**) Quantification of relative protein levels based on grayscale intensity. n = 3.

**(K-L).** Flow cytometry analysis of apoptosis in *HSPD1*-overexpressing (**K**) or -knockdown (**L**) cells following ALV-J infection. n = 4 (**K**) or n = 3(**L**).

For **B**, **C**, **E**, **F**, **H**, **J**, **K**, and **L**, the values are shown as the mean ± SD. *p < 0.05, **p < 0.01, ***p < 0.001 by unpaired Student’s t test.

**
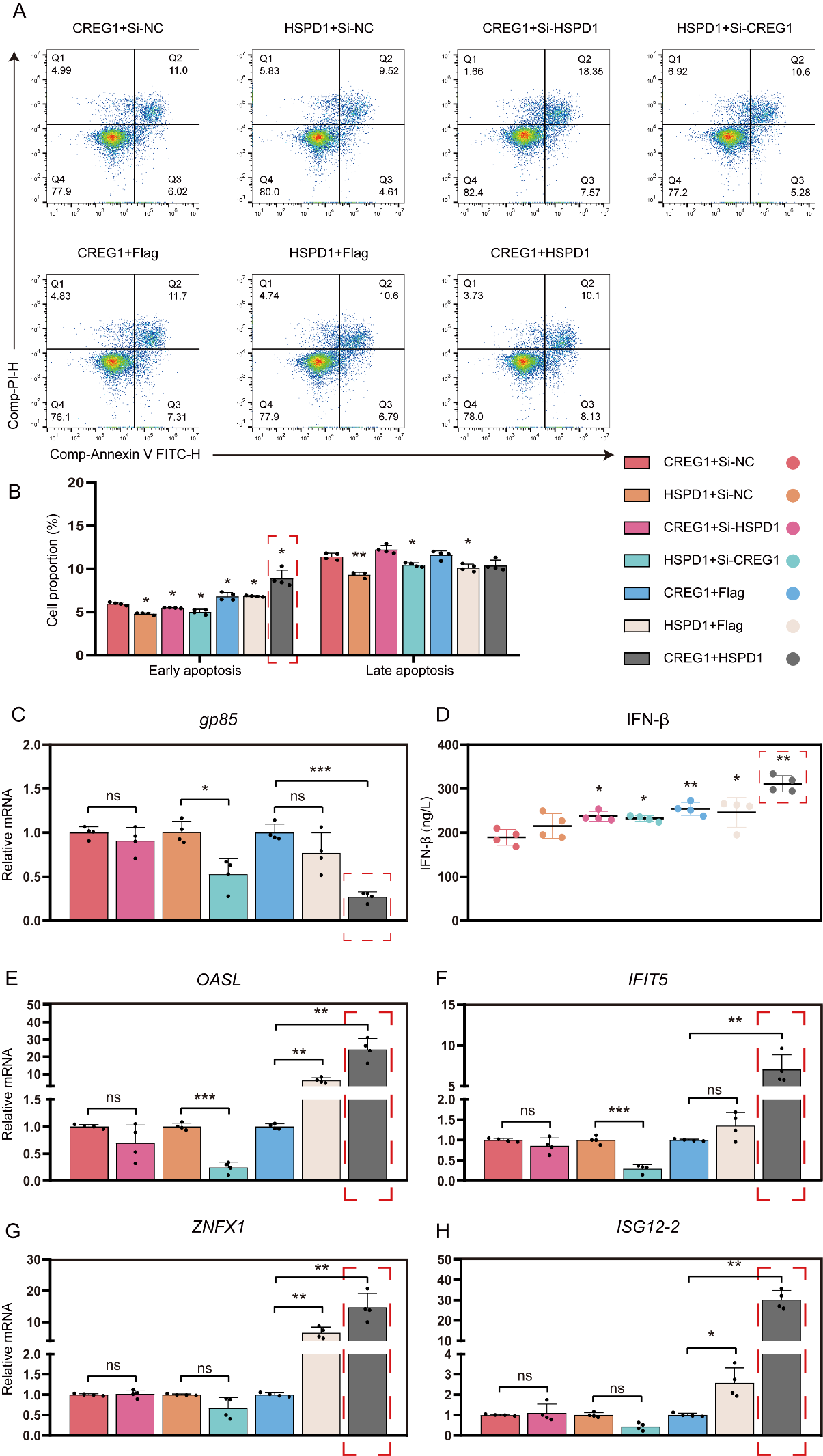
**

**Figure S8.** ***CREG1* and *HSPD1* cooperatively amplify antiviral responses.**

**(A-H).** In cells with *HSPD1* knockdown and *CREG1* overexpression, *CREG1* knockdown and *HSPD1* overexpression, or co-expression of *CREG1* and *HSPD1*, apoptosis was analyzed by flow cytometry (**A-B**), IFN-β levels in the supernatant were measured by ELISA (**D**), and gp85 (**C**) and ISGs (**E-H**) expression were assessed by RT-qPCR at 48 hpi following ALV-J infection. n = 4.

For **B**, all data were normalized relative to the *CREG1* + siNC group. For **B-D**, the data are presented as mean ±SD. Statistical significance was determined using unpaired Student’s t-test, with ns indicating no significant difference, *p < 0.05, **p < 0.01, and ***p < 0.001.
