# Supplementary material for: CREG1 restricts ALV-J replication via the mitochondrial dysfunction–driven activation of innate immunity and apoptosis
Source: Front Immunol. 2026 Jan 21;16:1760120. doi: 10.3389/fimmu.2025.1760120 (PMC12867876; doi:10.3389/fimmu.2025.1760120)

Uncropped western blot images
Figure 1

1F

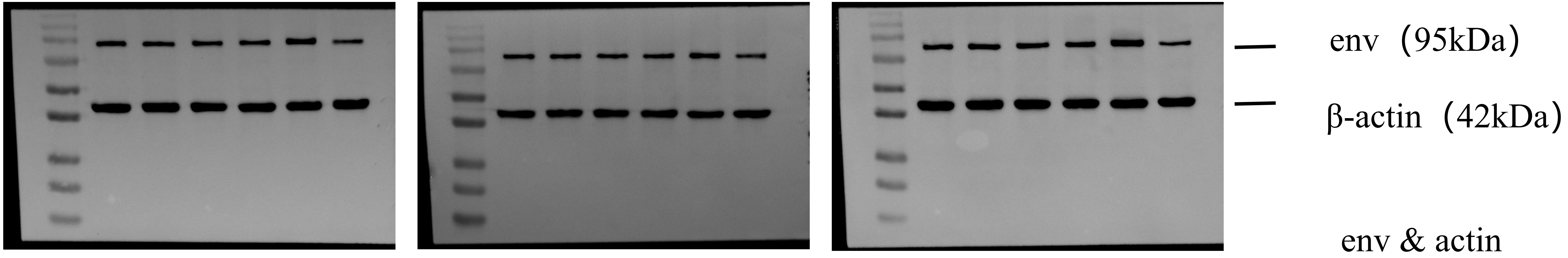

1I

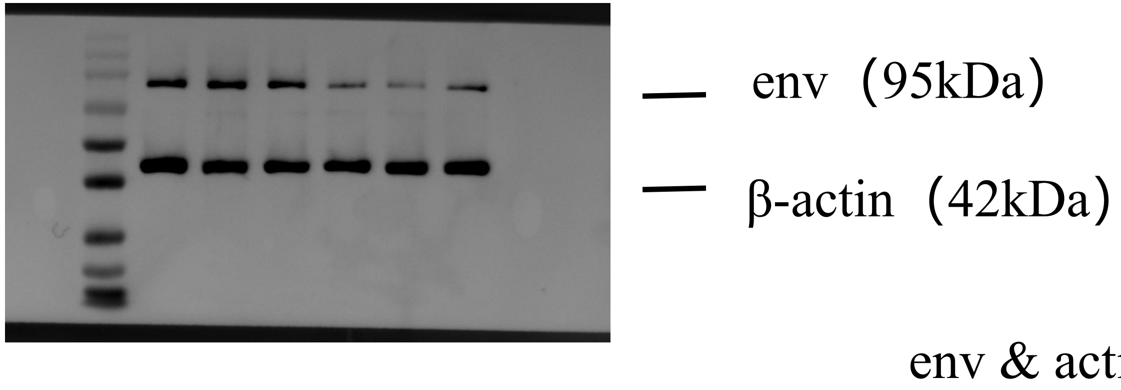

Figure 2

2G

β-actin (42kDa)

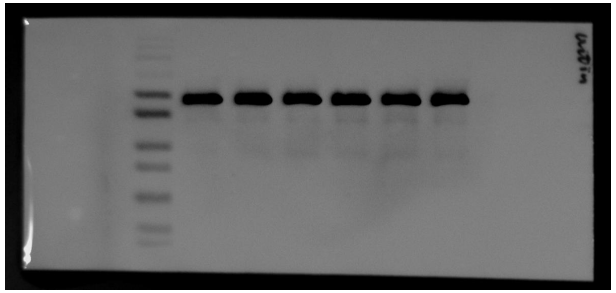

JAK1 (130kDa)

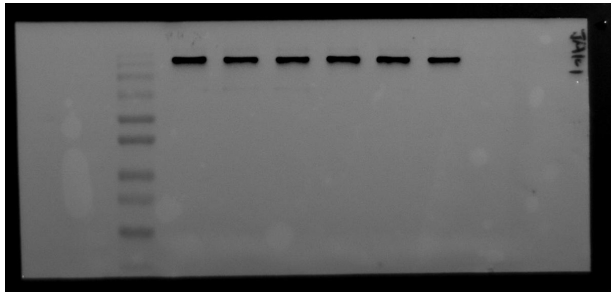

P-JAK1 (130kDa)

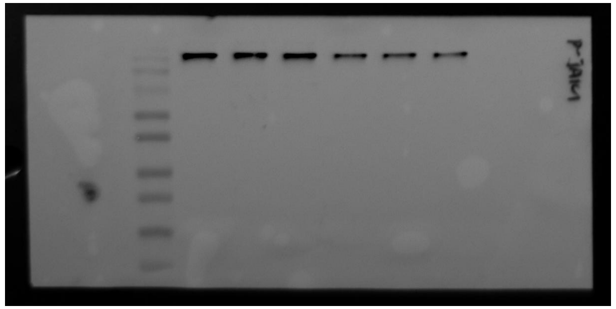

P-STAT1 (91kDa)

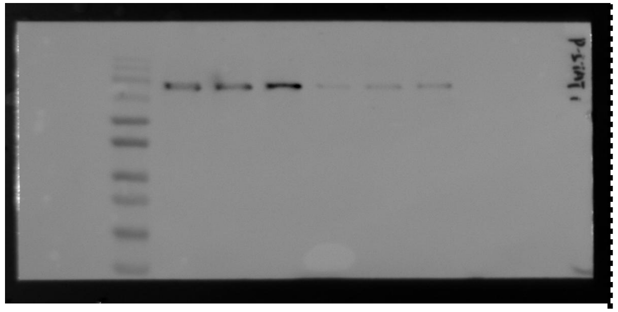

P-TBK1 (84kDa)

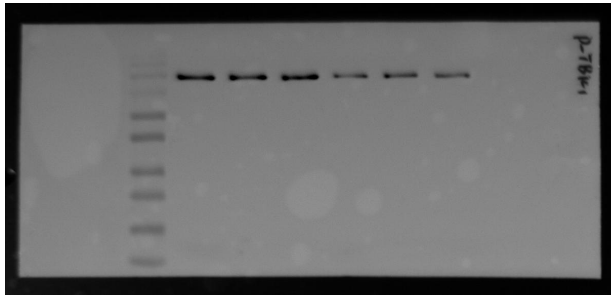

STAT1 (83kDa)

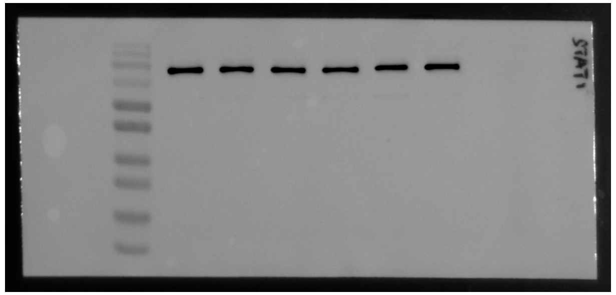

TBK1 (84kDa)

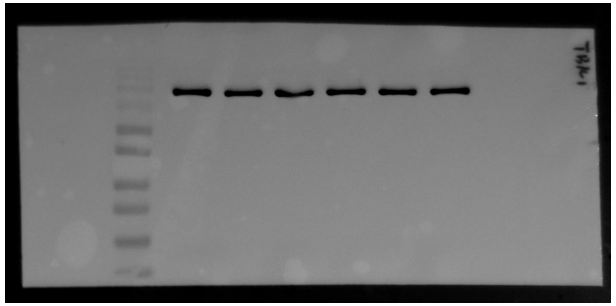

2I

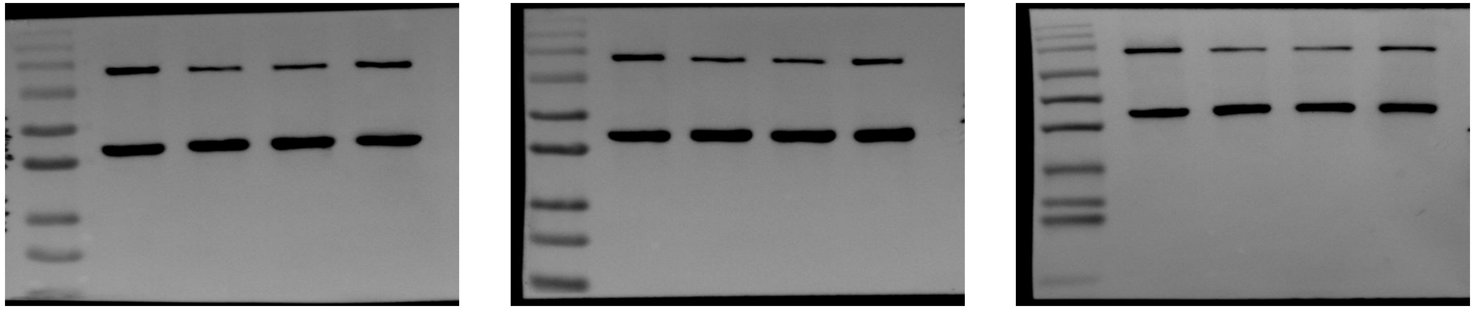

env (95kDa)

actin (42kDa)

env & actin

Figure 2

2J

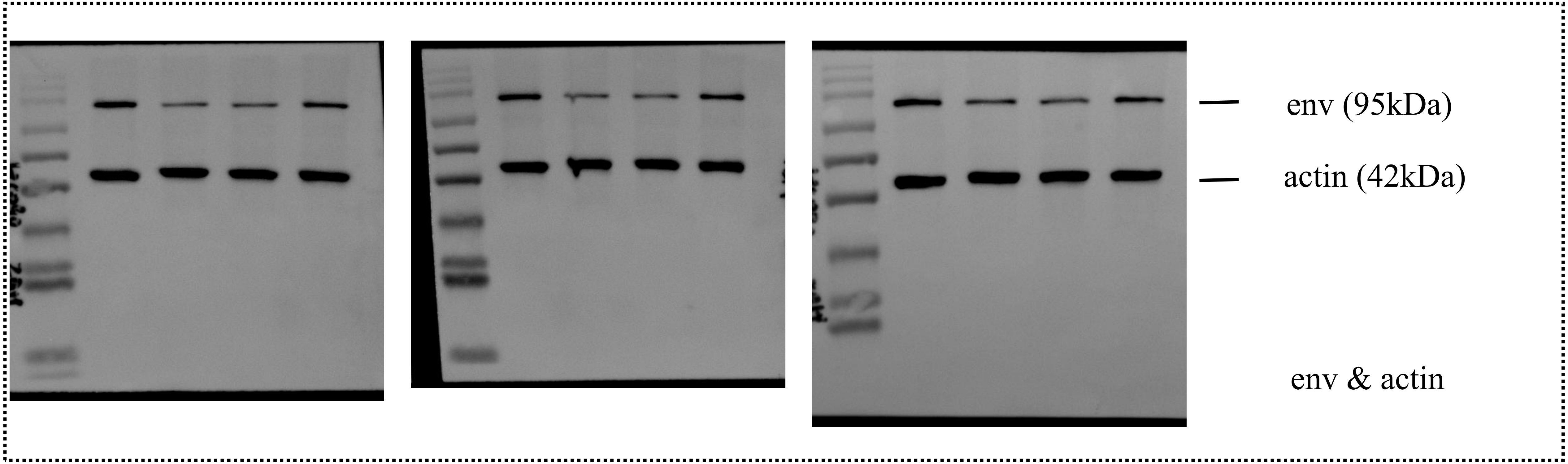

Figure 4

4A cytoplasm

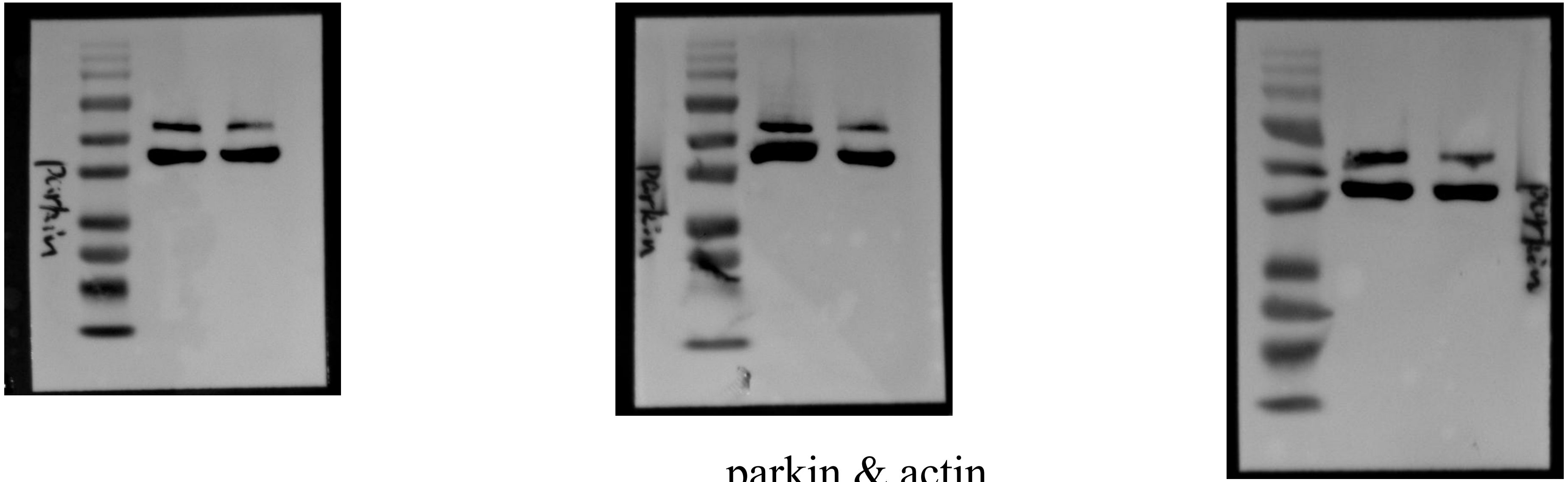

parkin (52kDa)
actin (42kDa)

parkin & actin

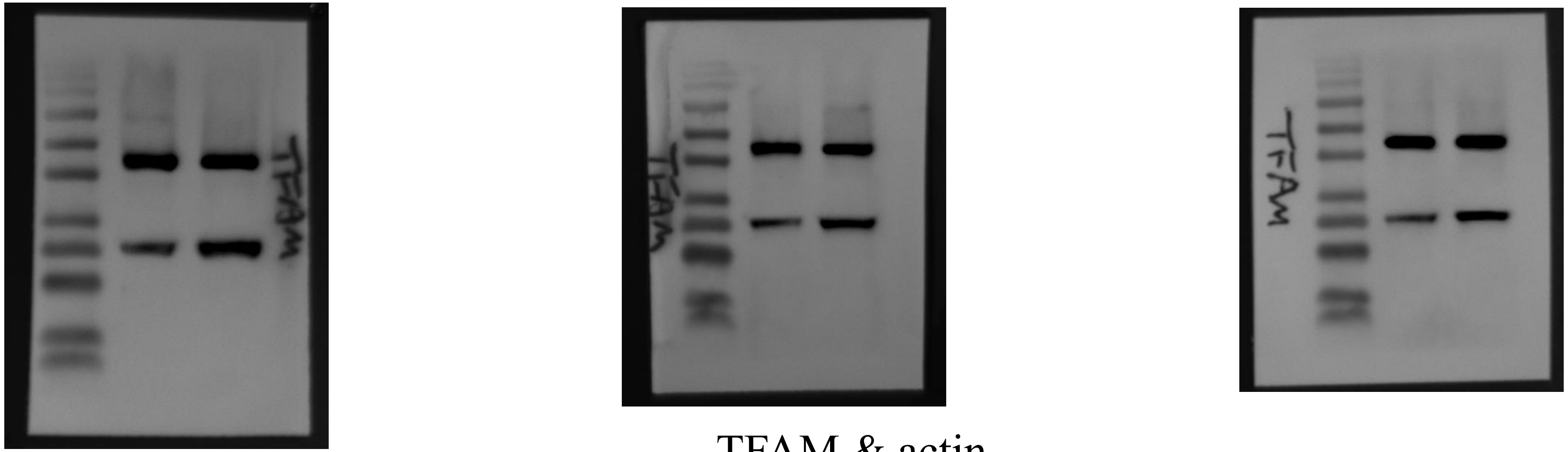

actin (42kDa)
TFAM (25kDa)

TFAM & actin

Figure 4

4A mitochondria

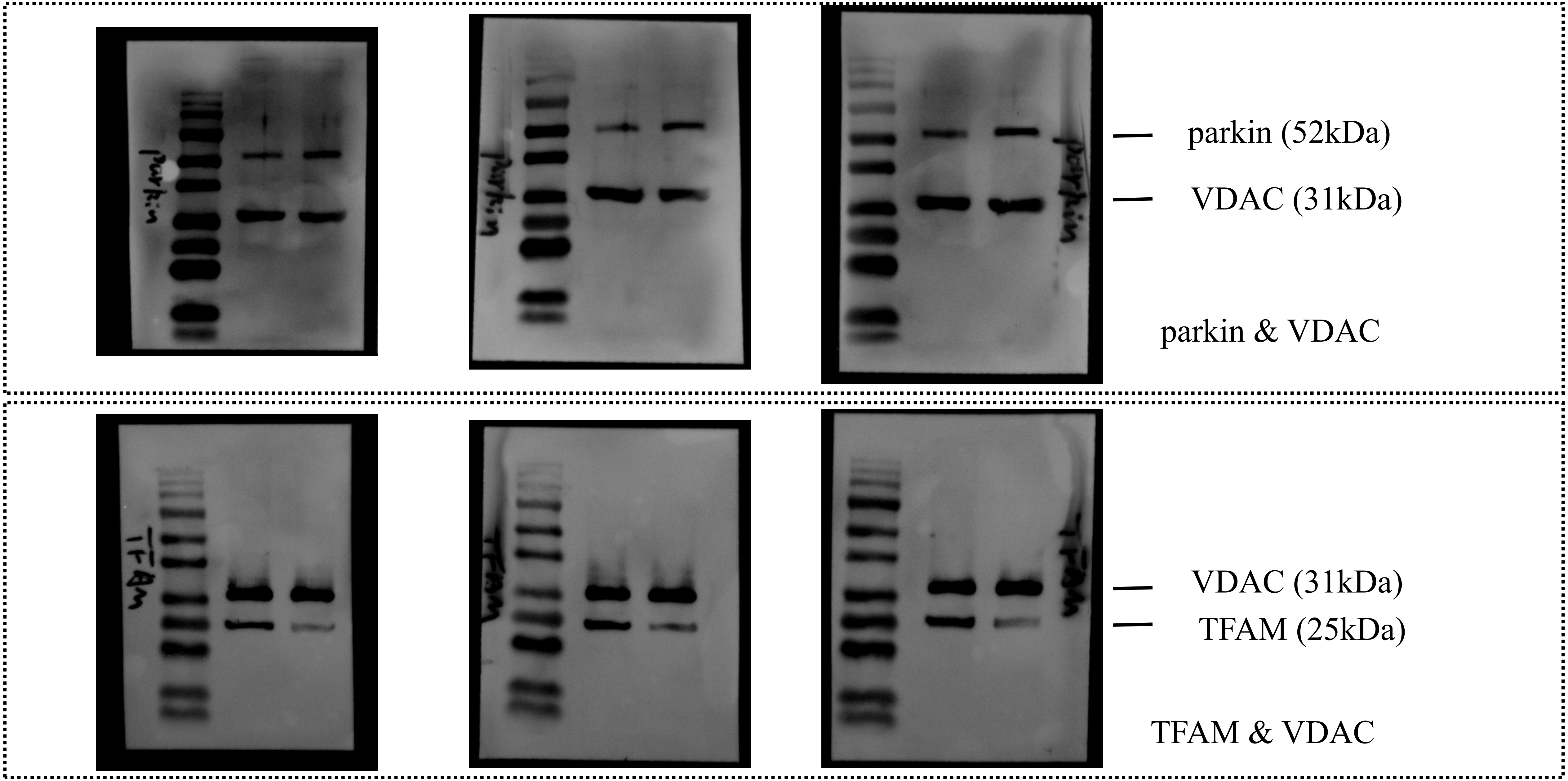

Figure 4

4A mitochondria

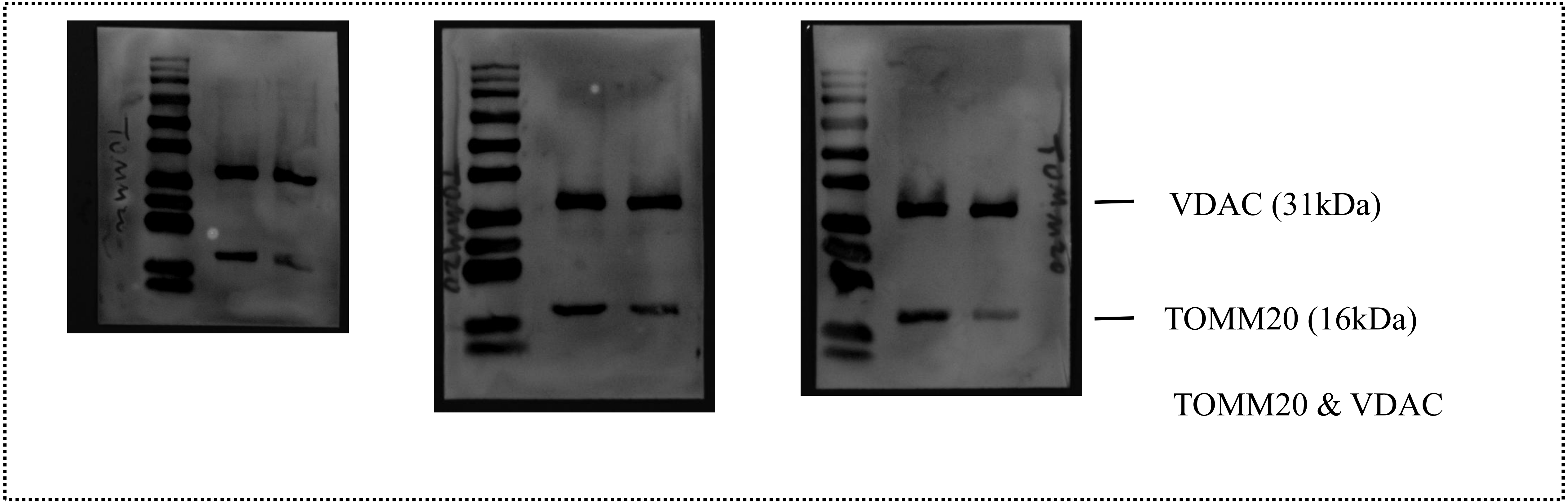

Figure 4

4C cytoplasm

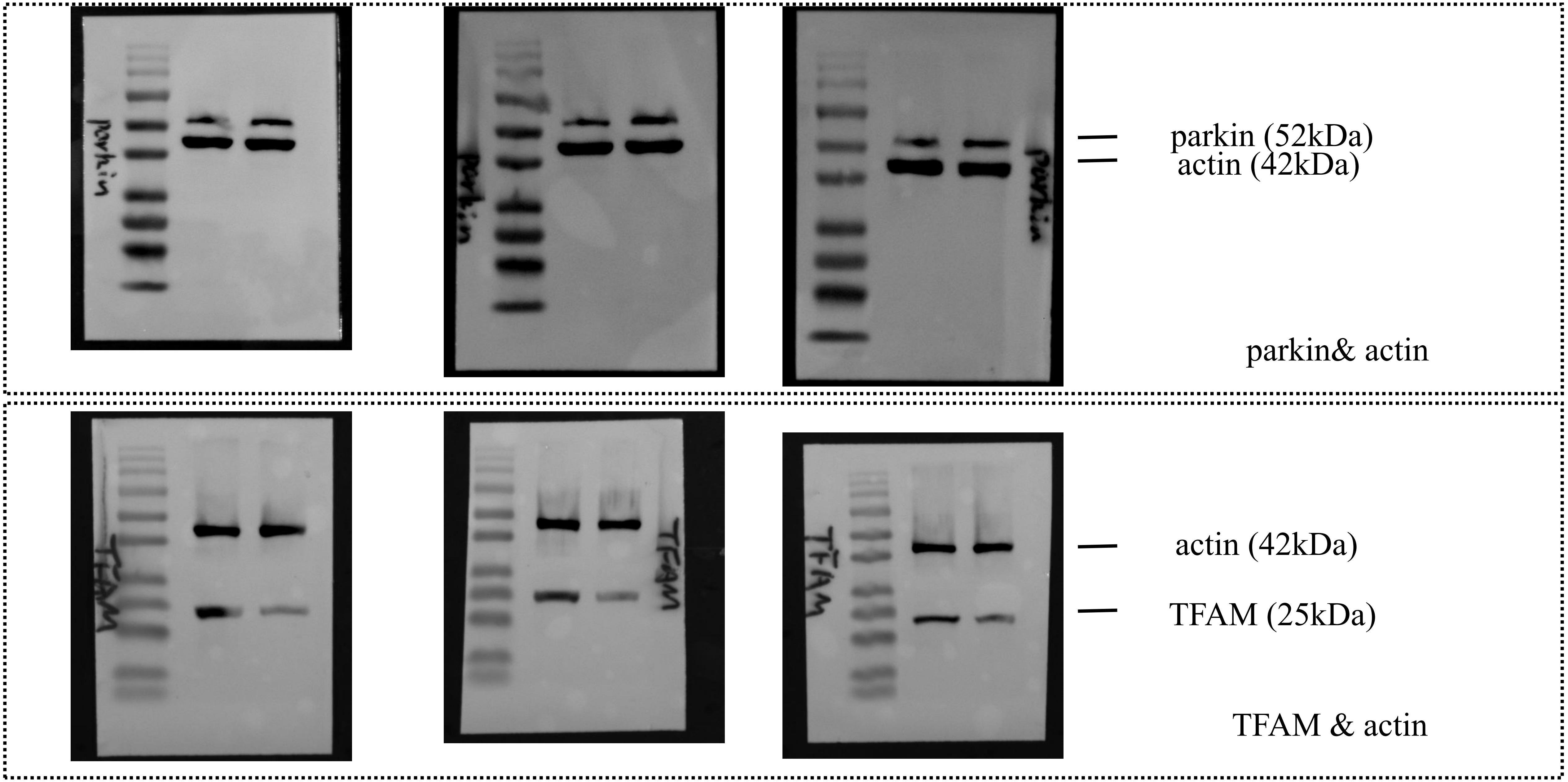

Figure 4

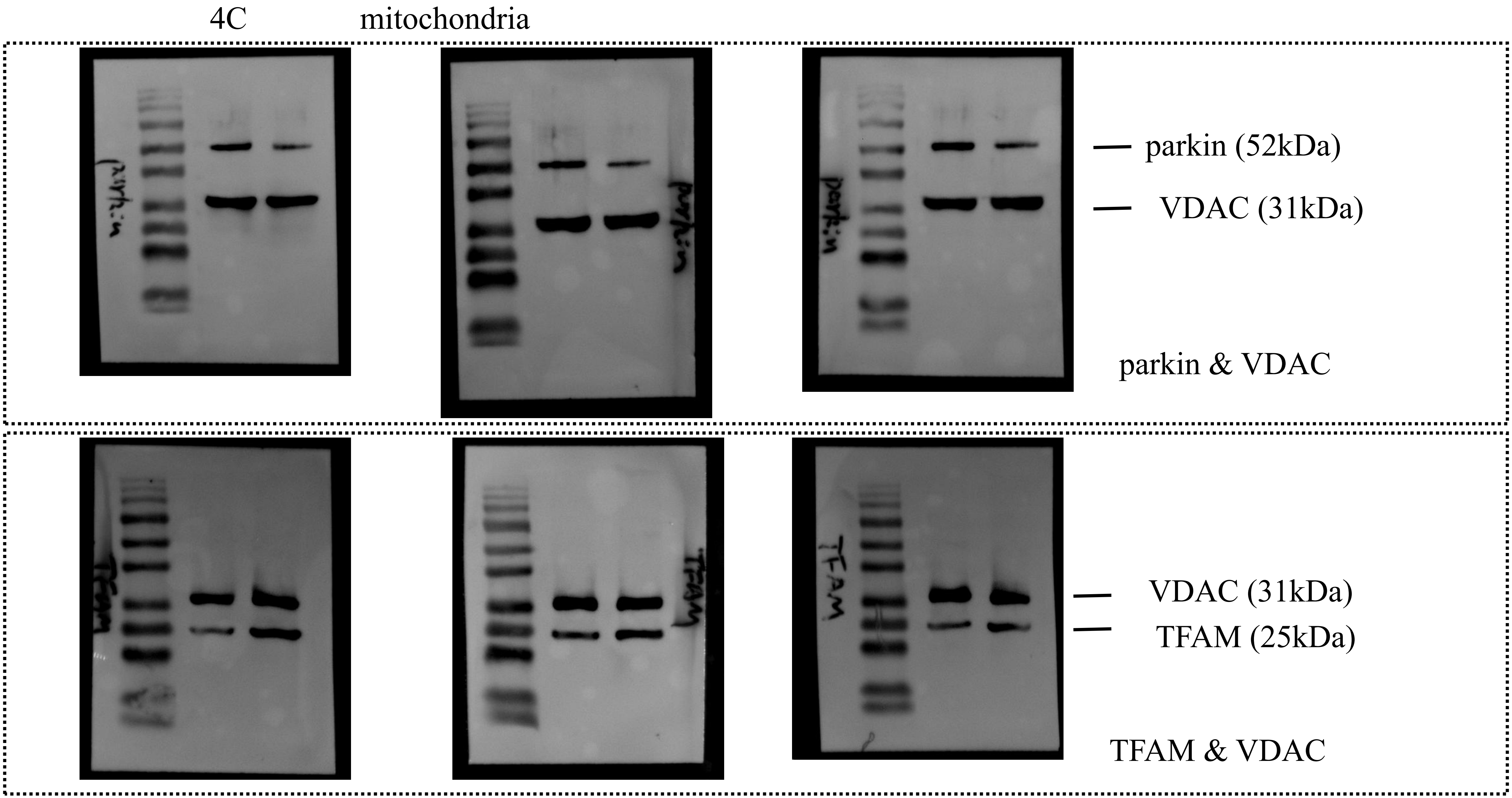
4C
mitochondria
parkin (52kDa)
VDAC (31kDa)
parkin & VDAC
VDAC (31kDa)
TFAM (25kDa)
TFAM & VDAC

Figure 4

4C mitochondria

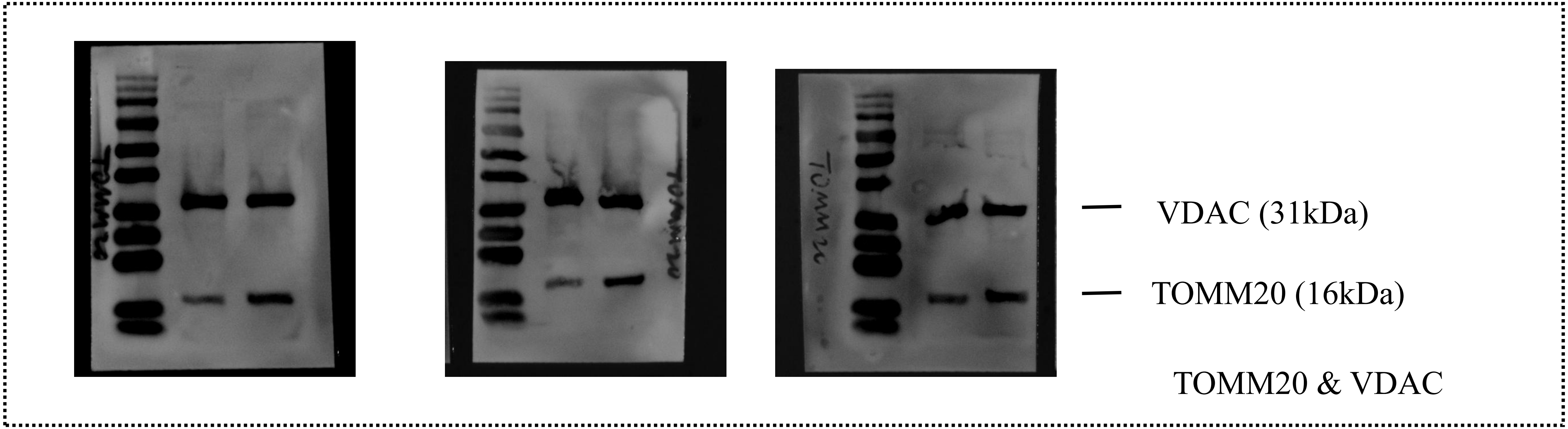

Figure 4

4B

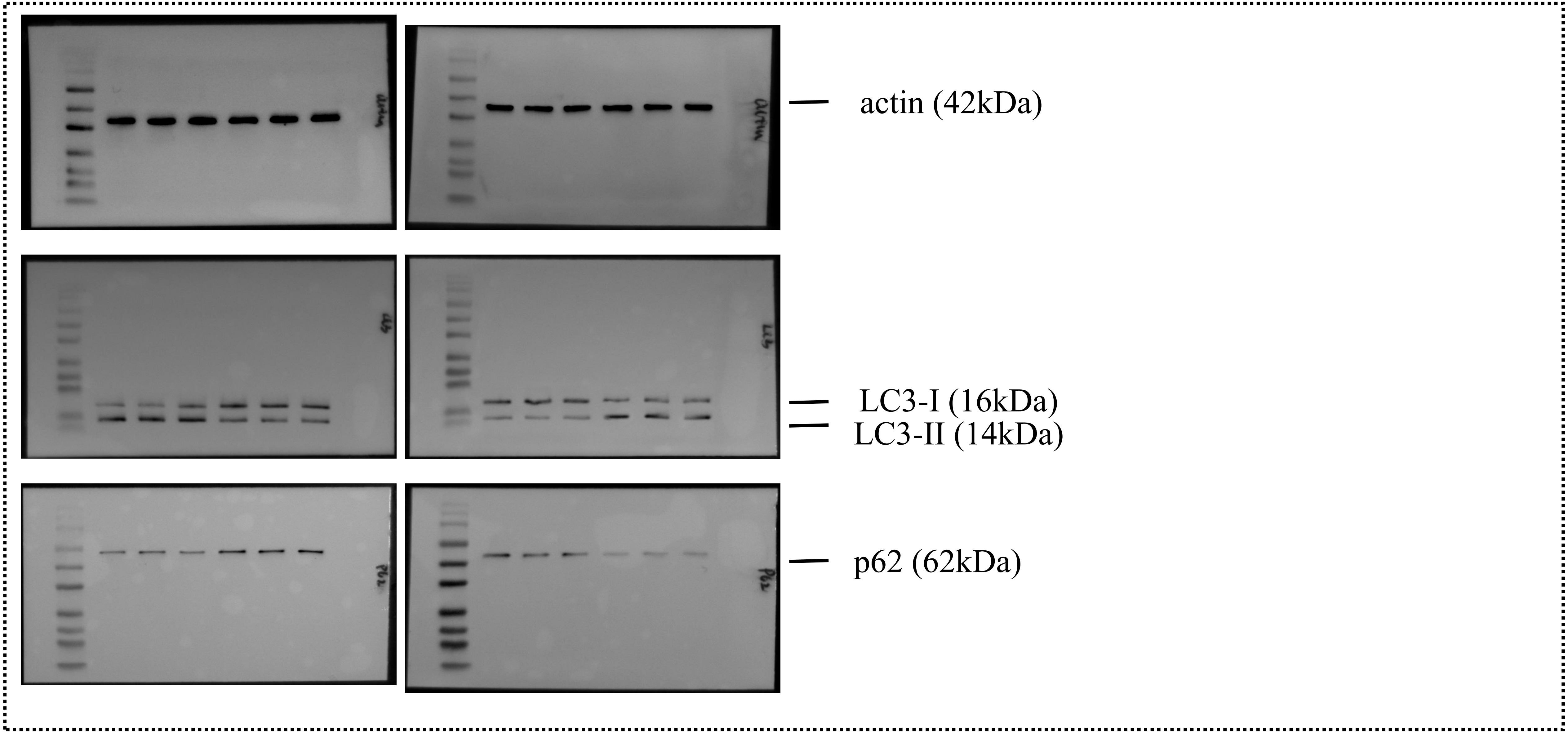

Figure 5

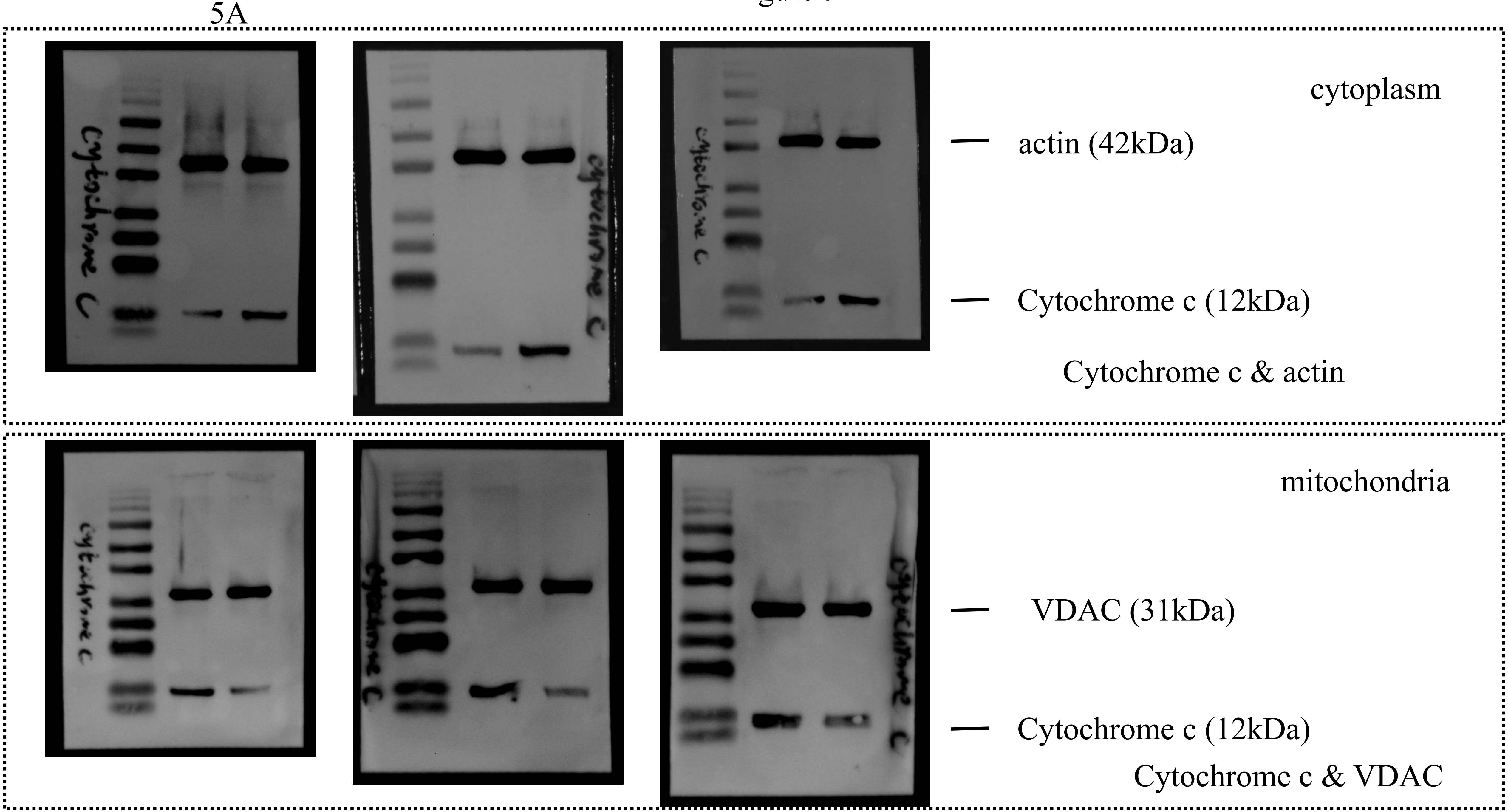
5A
cytoplasm
actin (42kDa)
Cytochrome c (12kDa)
Cytochrome c & actin
mitochondria
VDAC (31kDa)
Cytochrome c (12kDa)
Cytochrome c & VDAC

Figure 5

5C

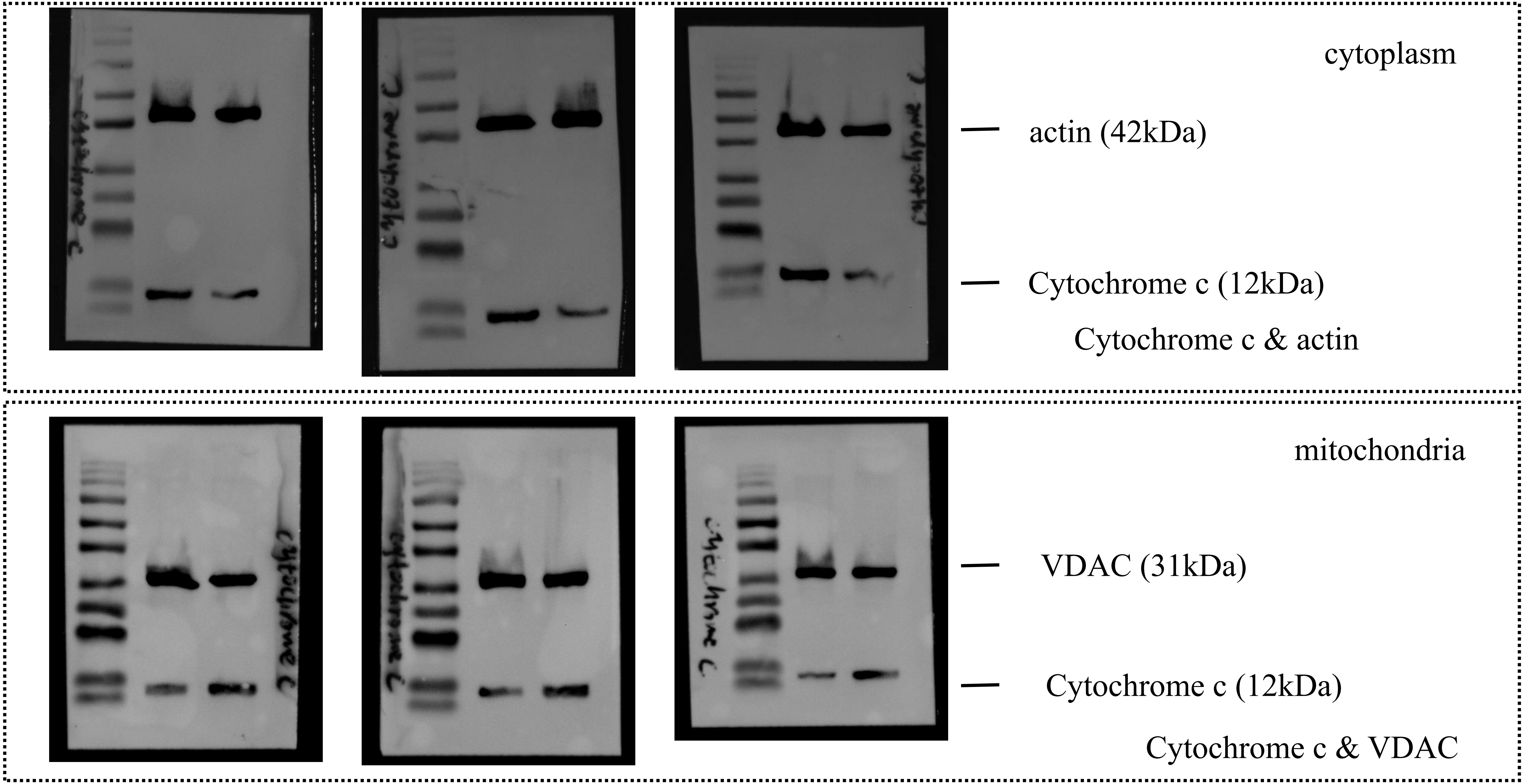
cytoplasm
actin (42kDa)
Cytochrome c (12kDa)
Cytochrome c & actin
mitochondria
VDAC (31kDa)
Cytochrome c (12kDa)
Cytochrome c & VDAC

Figure 5

5G

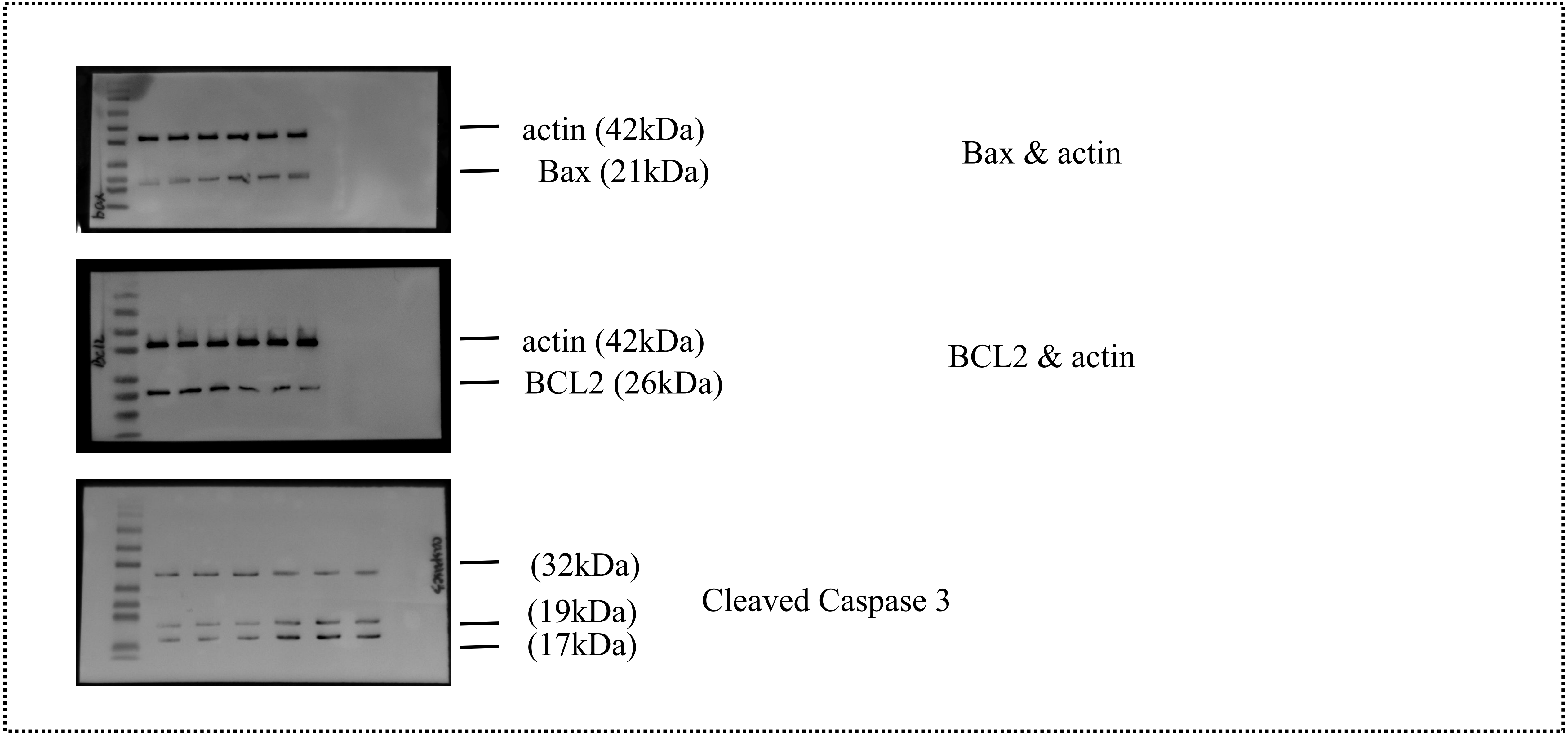
actin (42kDa)
Bax (21kDa)
Bax & actin
actin (42kDa)
BCL2 (26kDa)
BCL2 & actin
(32kDa)
(19kDa)
(17kDa)
Cleaved Caspase 3

Figure 5

5K

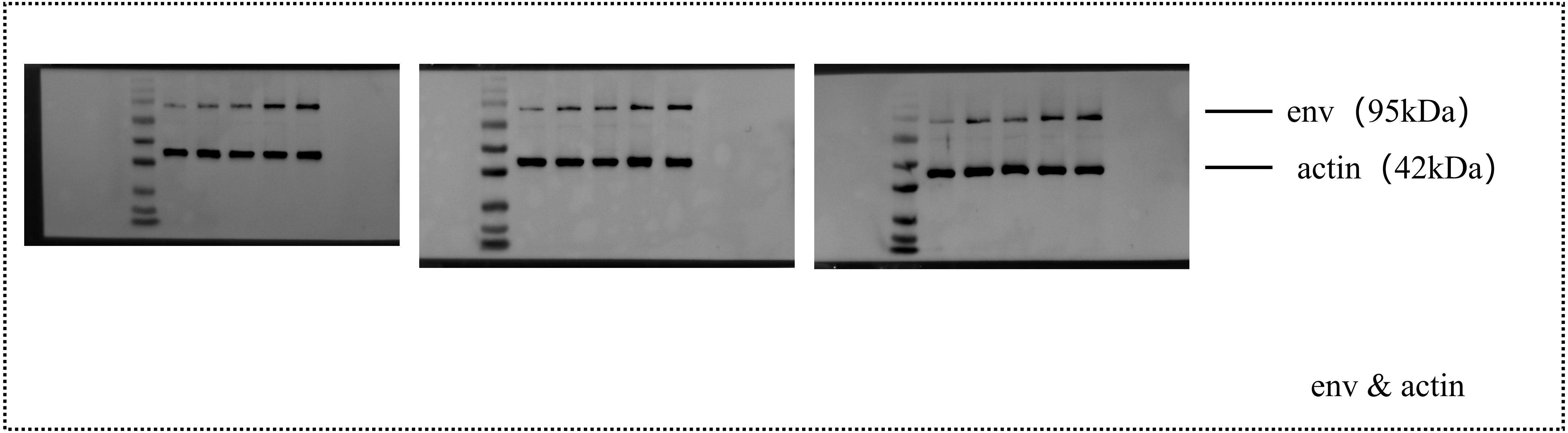

Figure 6

CO-IP for mass spectrometry

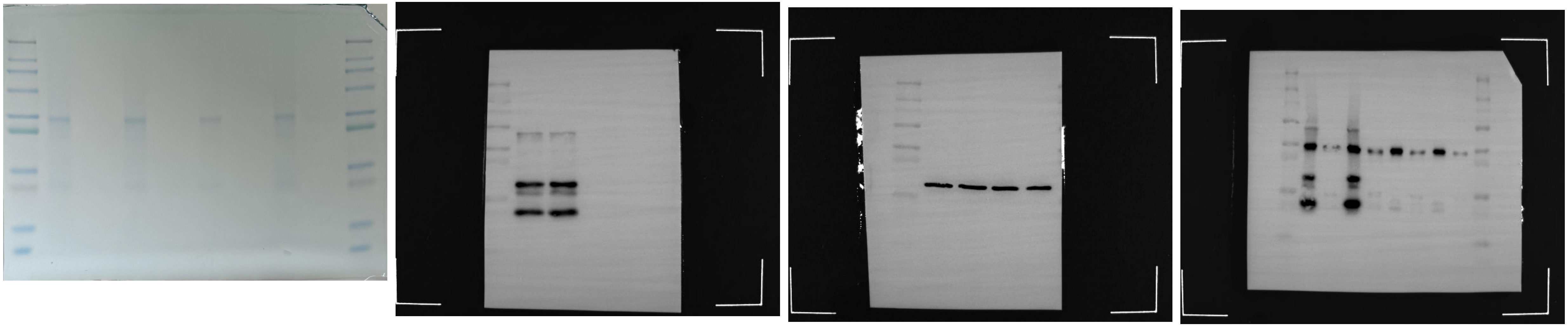

Coomassie brilliant blue

IB-Flag-CREG1

GAPDH

IP-Flag

Figure 6

6A-6B

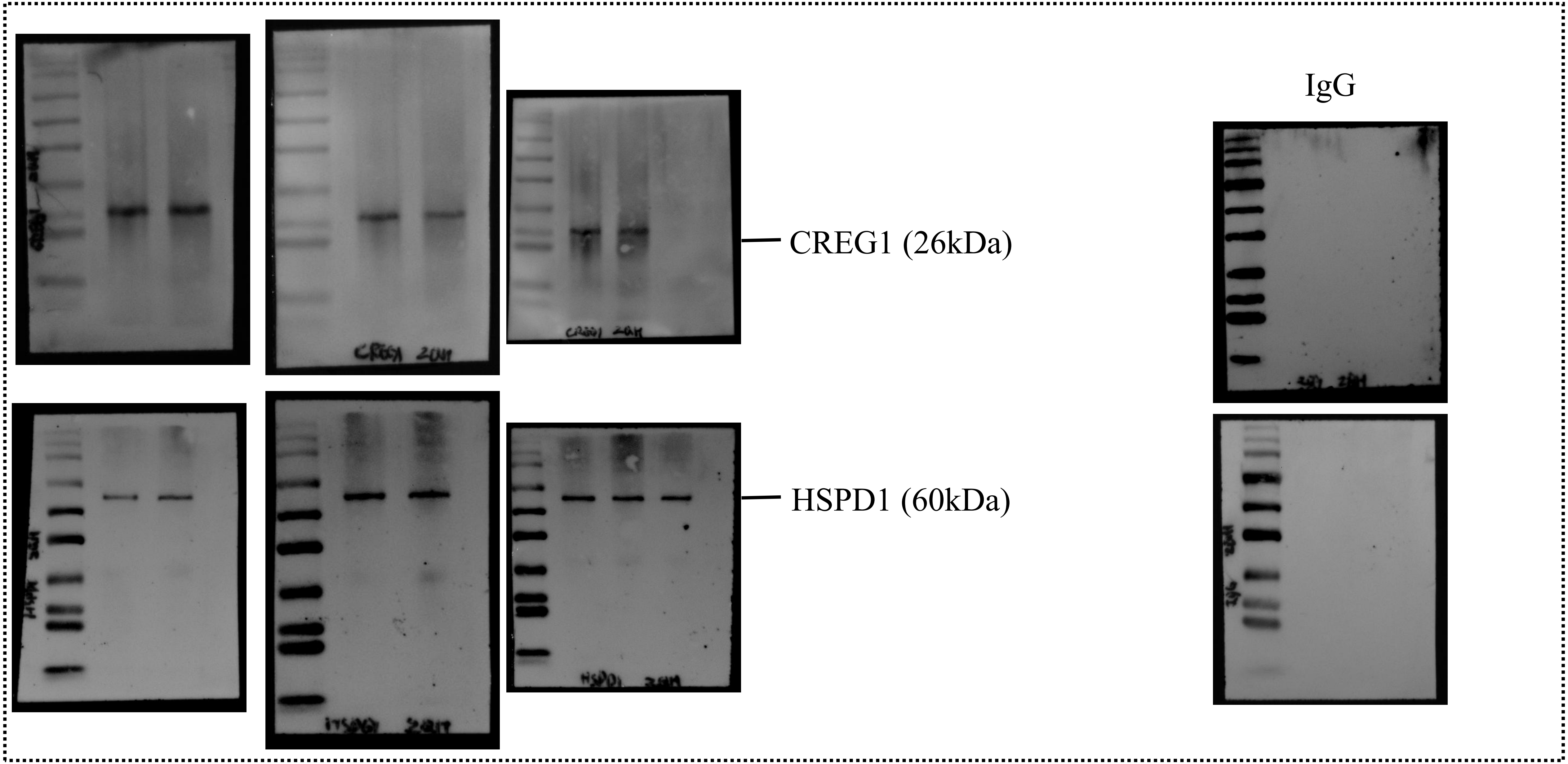

Figure 6

6C

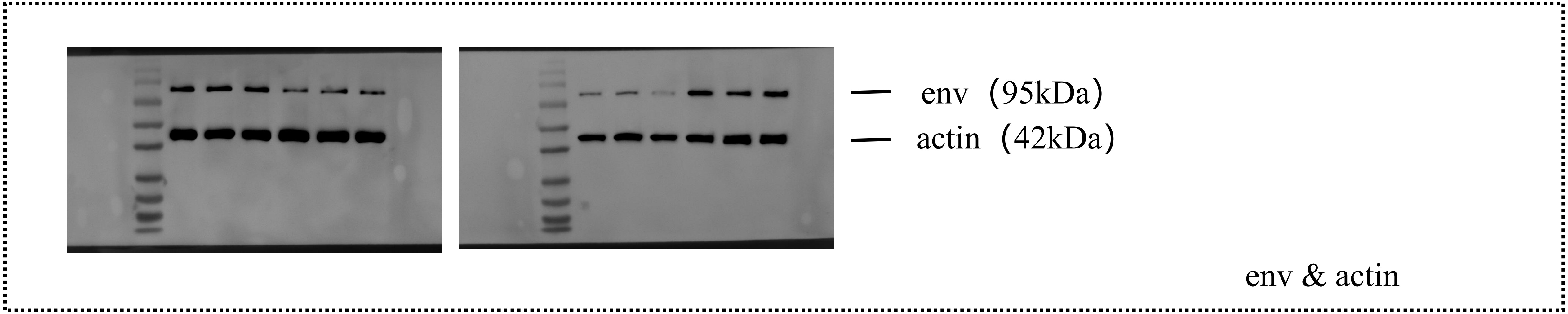

Figure 6

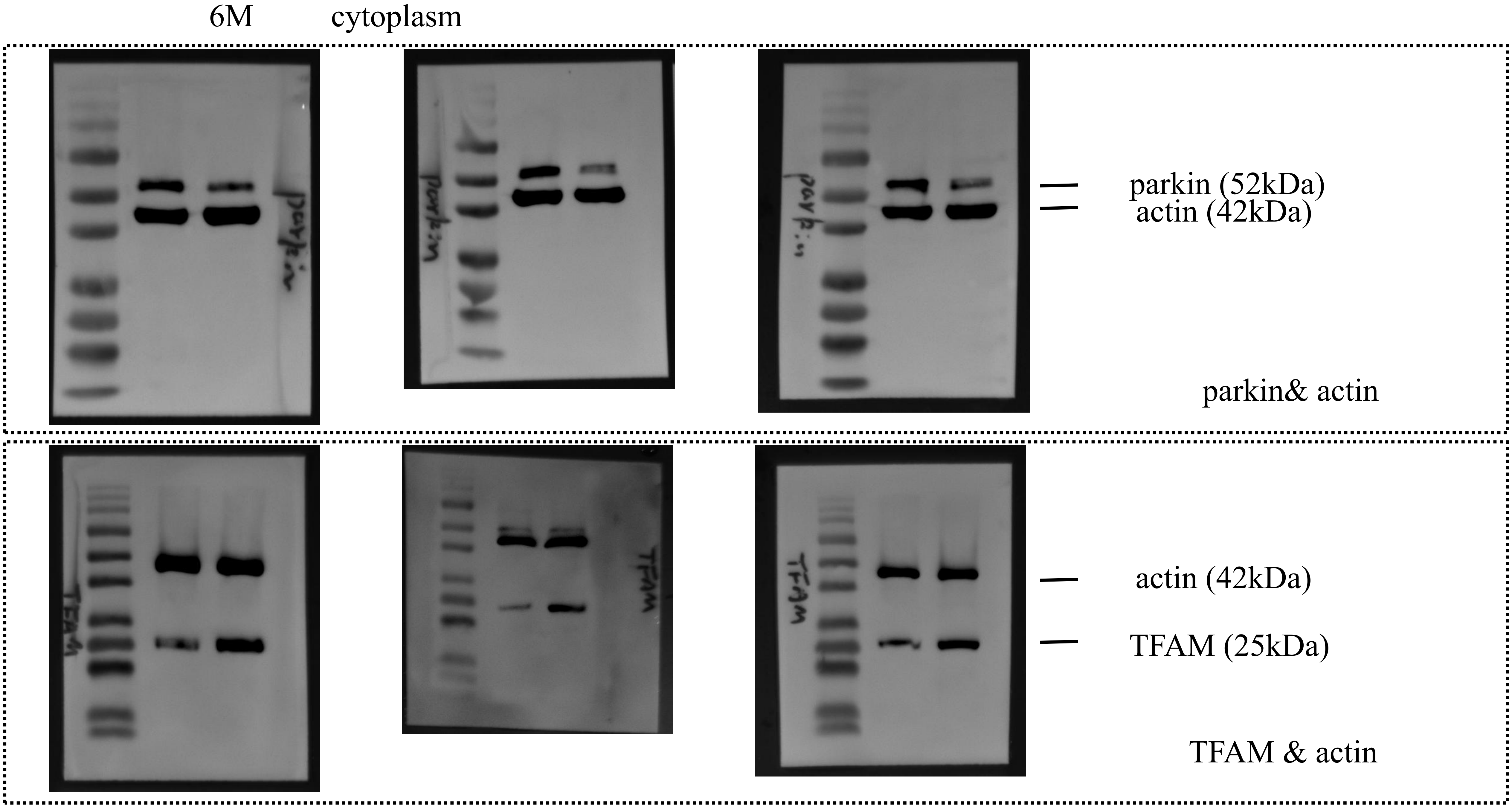
6M
cytoplasm
parkin (52kDa)
actin (42kDa)
parkin& actin
actin (42kDa)
TFAM (25kDa)
TFAM & actin

Figure 6

6M cytoplasm

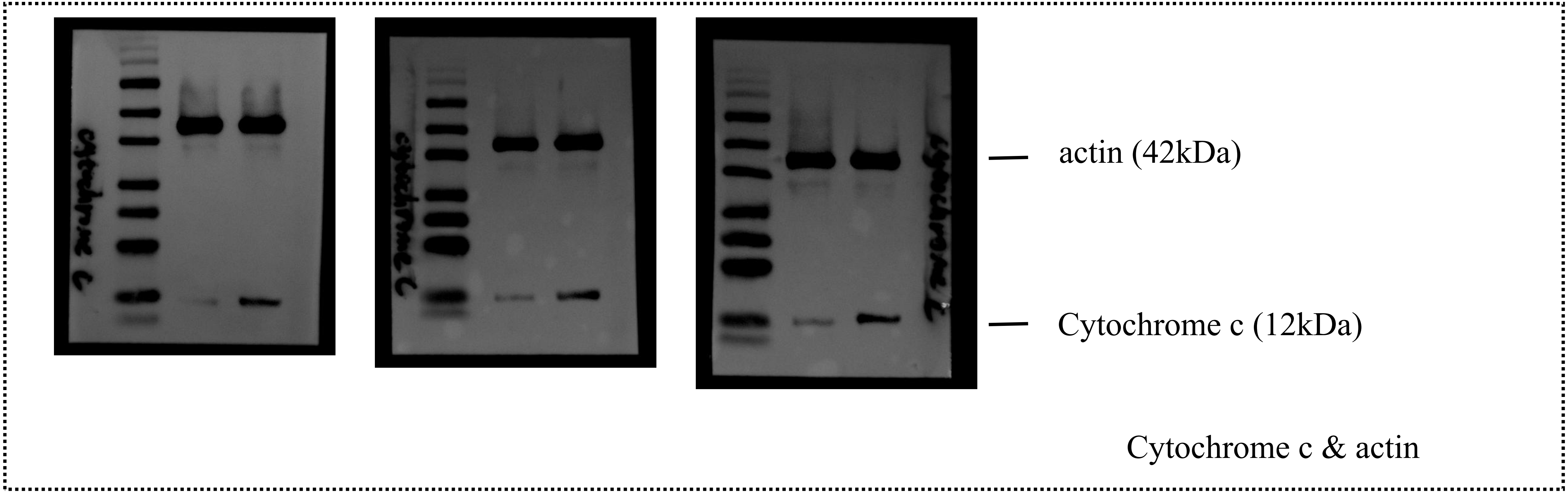

Figure 6

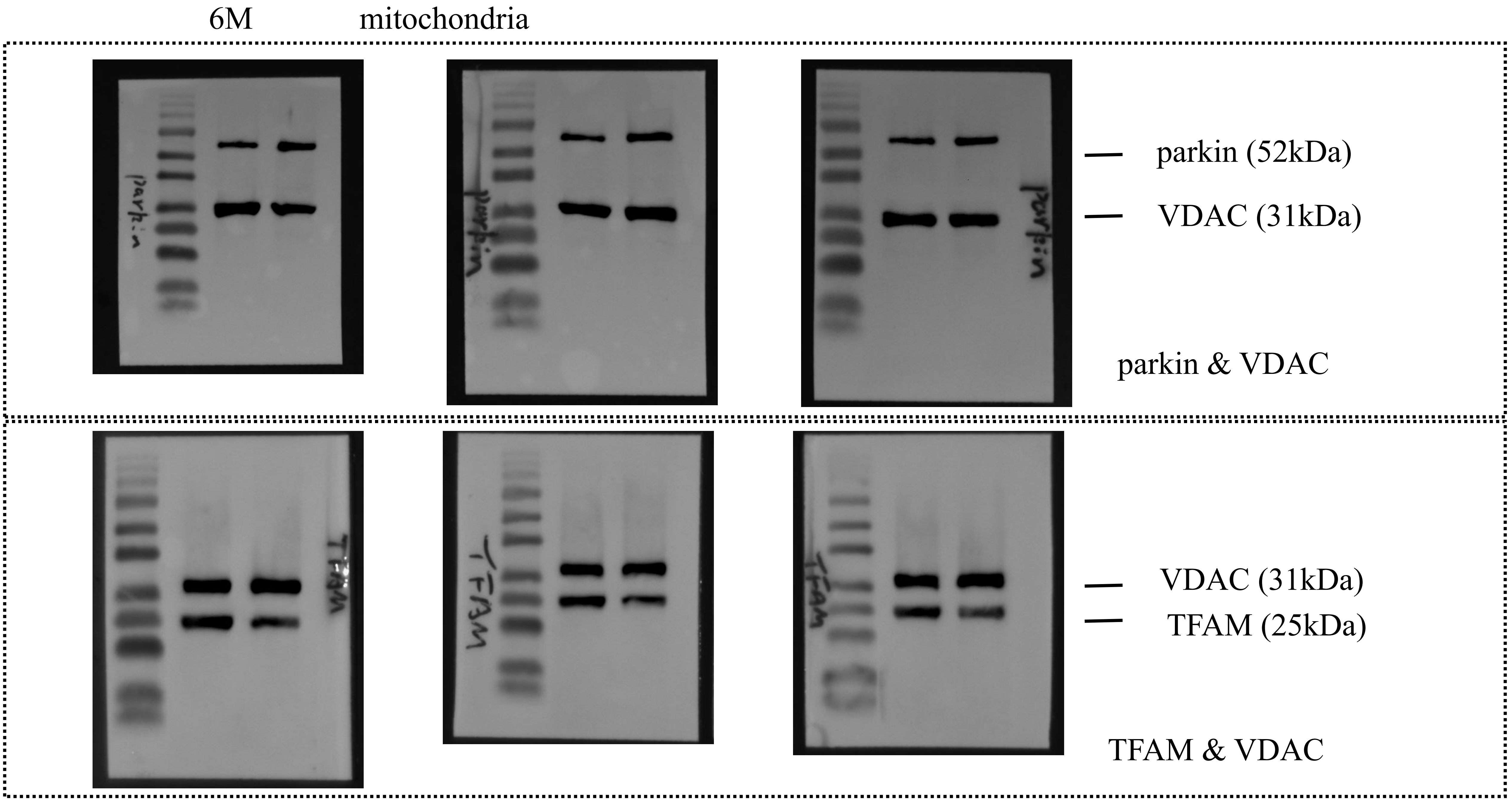
6M
mitochondria
parkin (52kDa)
VDAC (31kDa)
parkin & VDAC
VDAC (31kDa)
TFAM (25kDa)
TFAM & VDAC

Figure 6

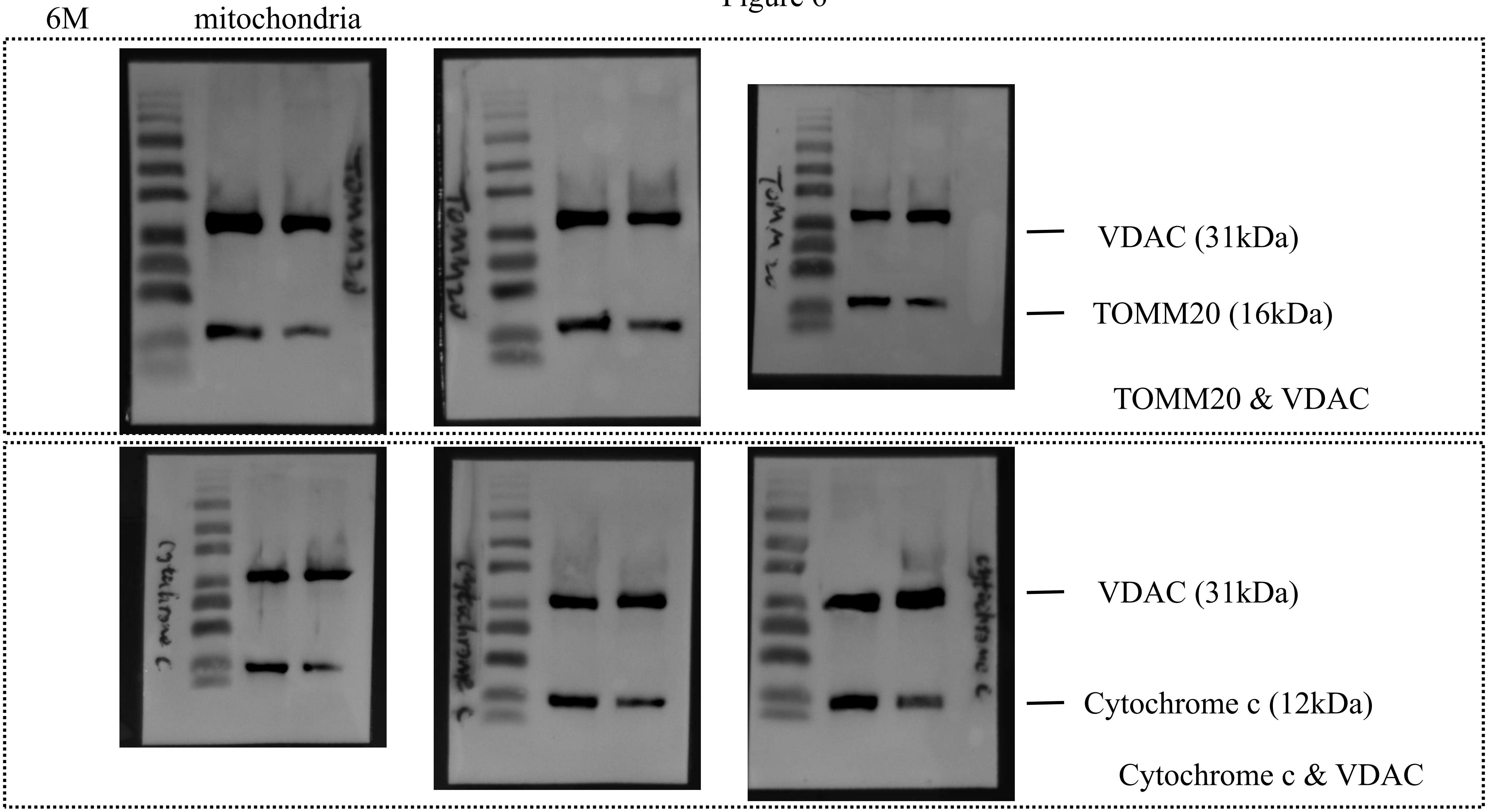
6M
mitochondria
VDAC (31kDa)
TOMM20 (16kDa)
TOMM20 & VDAC
VDAC (31kDa)
Cytochrome c (12kDa)
Cytochrome c & VDAC

Figure S 7

S7I

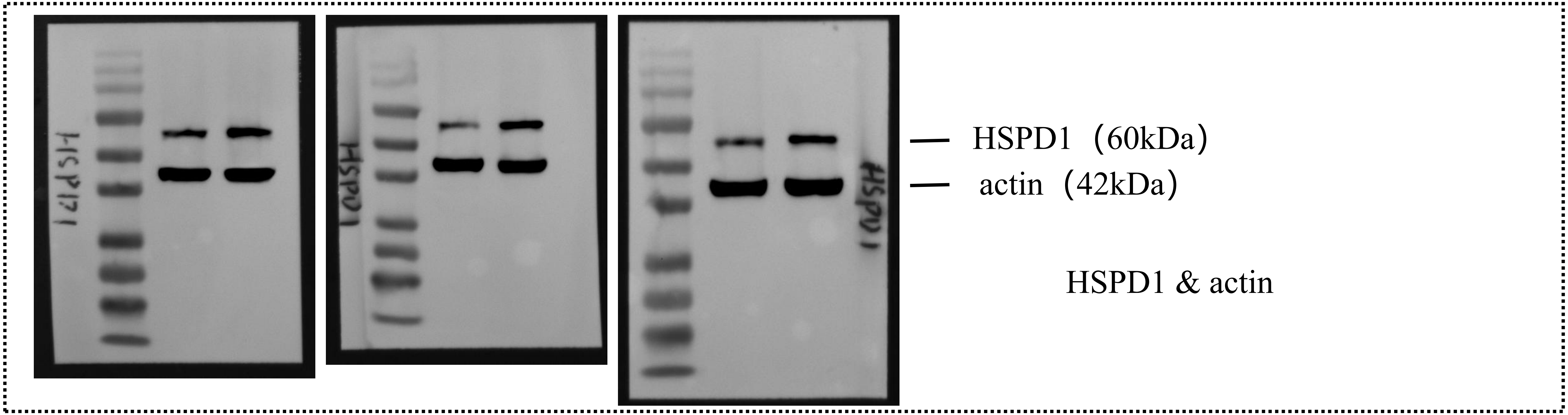

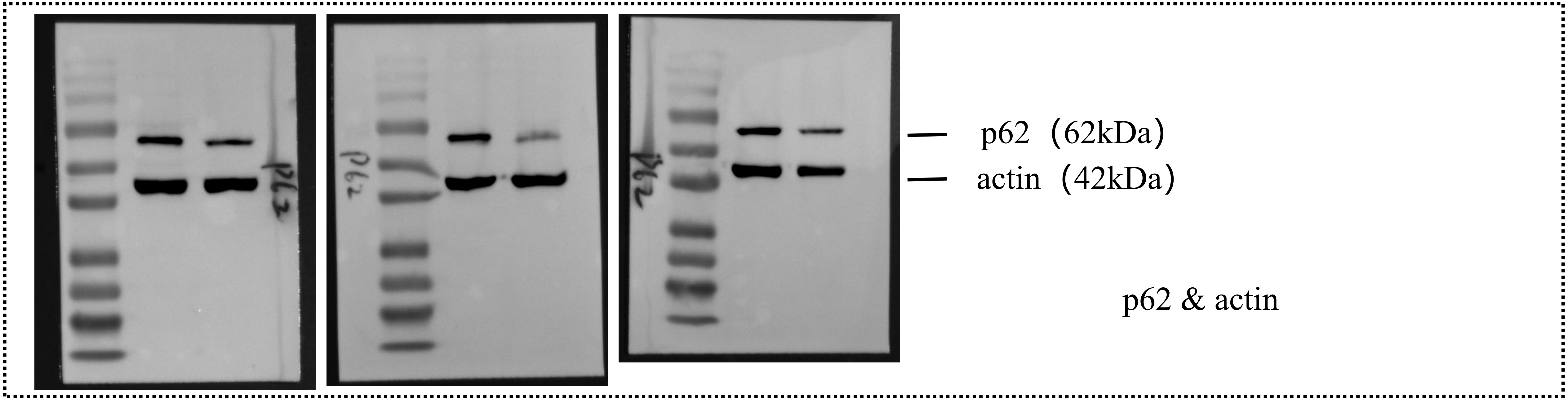

Figure 7

7A

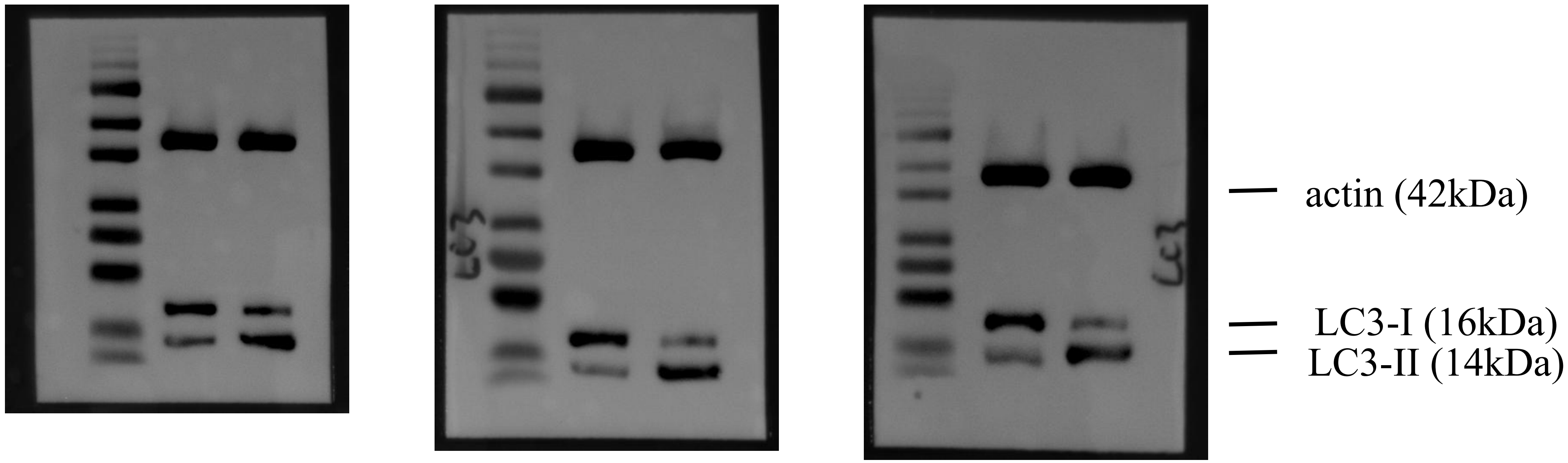

LC3-I/II & actin

Figure 7

7G

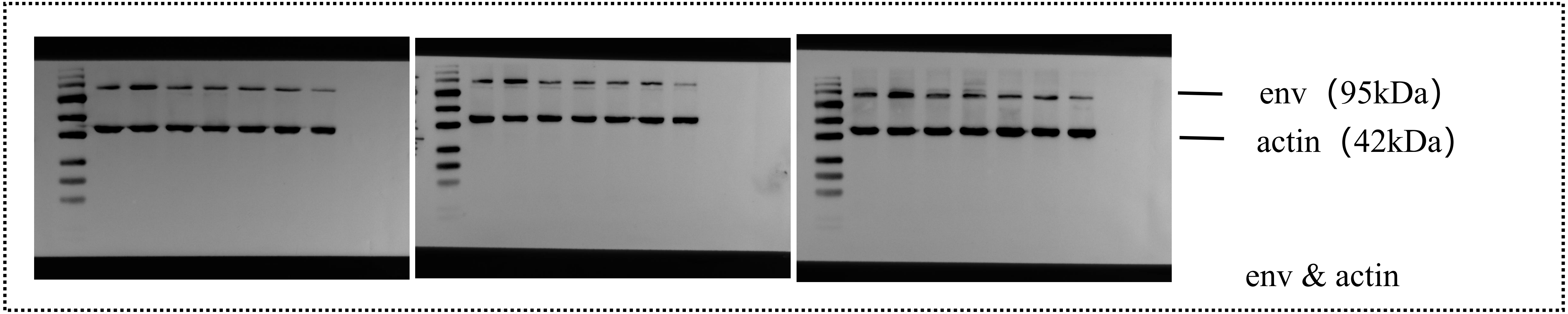

Figure S3

S2C

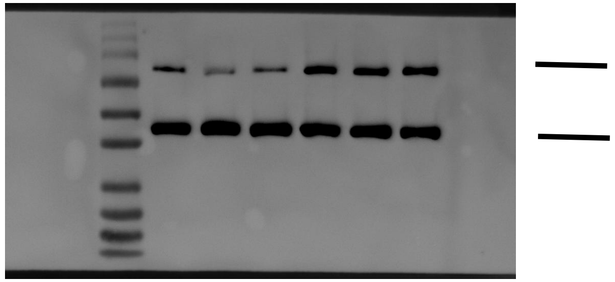

env & actin

Figure S3

S3C

β-actin (42kDa)

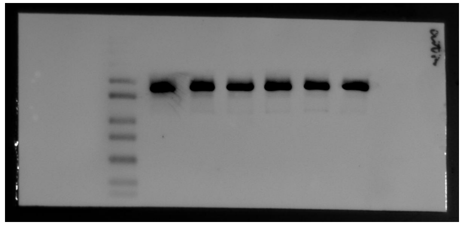

JAK1 (130kDa)

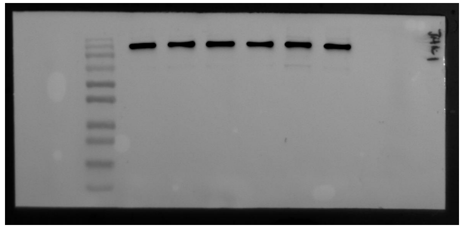

P-JAK1 (130kDa)

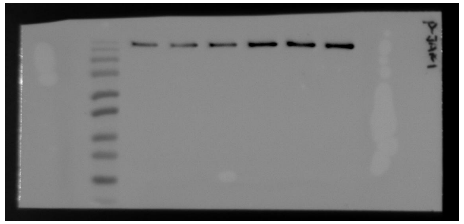

P-STAT1 (91kDa)

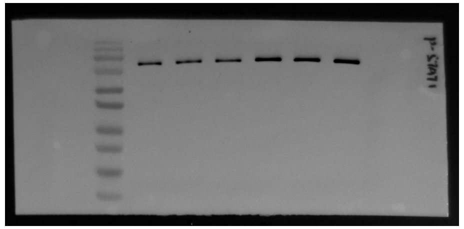

P-TBK1 (84kDa)

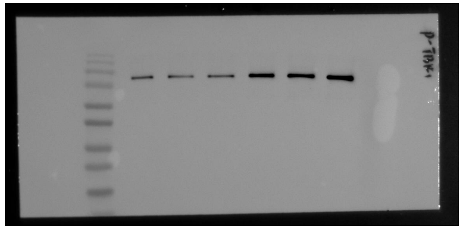

STAT1 (83kDa)

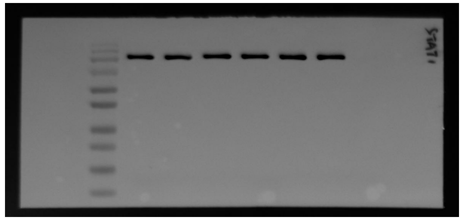

TBK1 (84kDa)

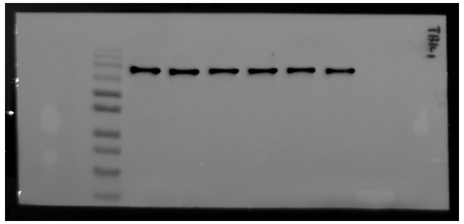

Figure S5

S5G

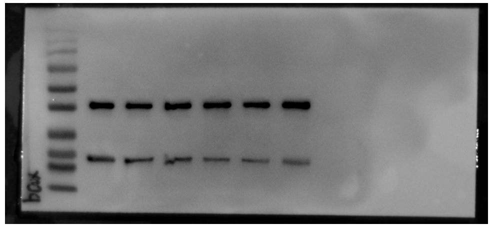

actin (42kDa)

Bax (21kDa)

Bax & actin

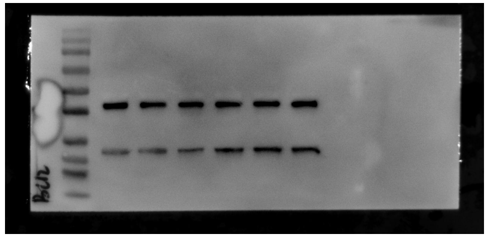

actin (42kDa)

BCL2 (26kDa)

BCL2 & actin

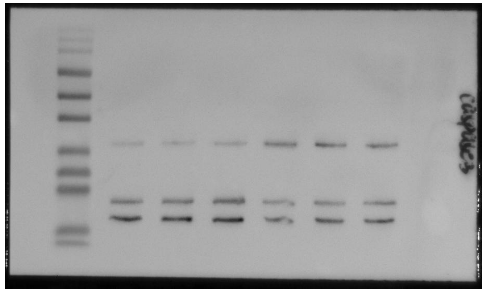

(32kDa)

(19kDa)

(17kDa)

Cleaved Caspase 3

Figure S7

S8A

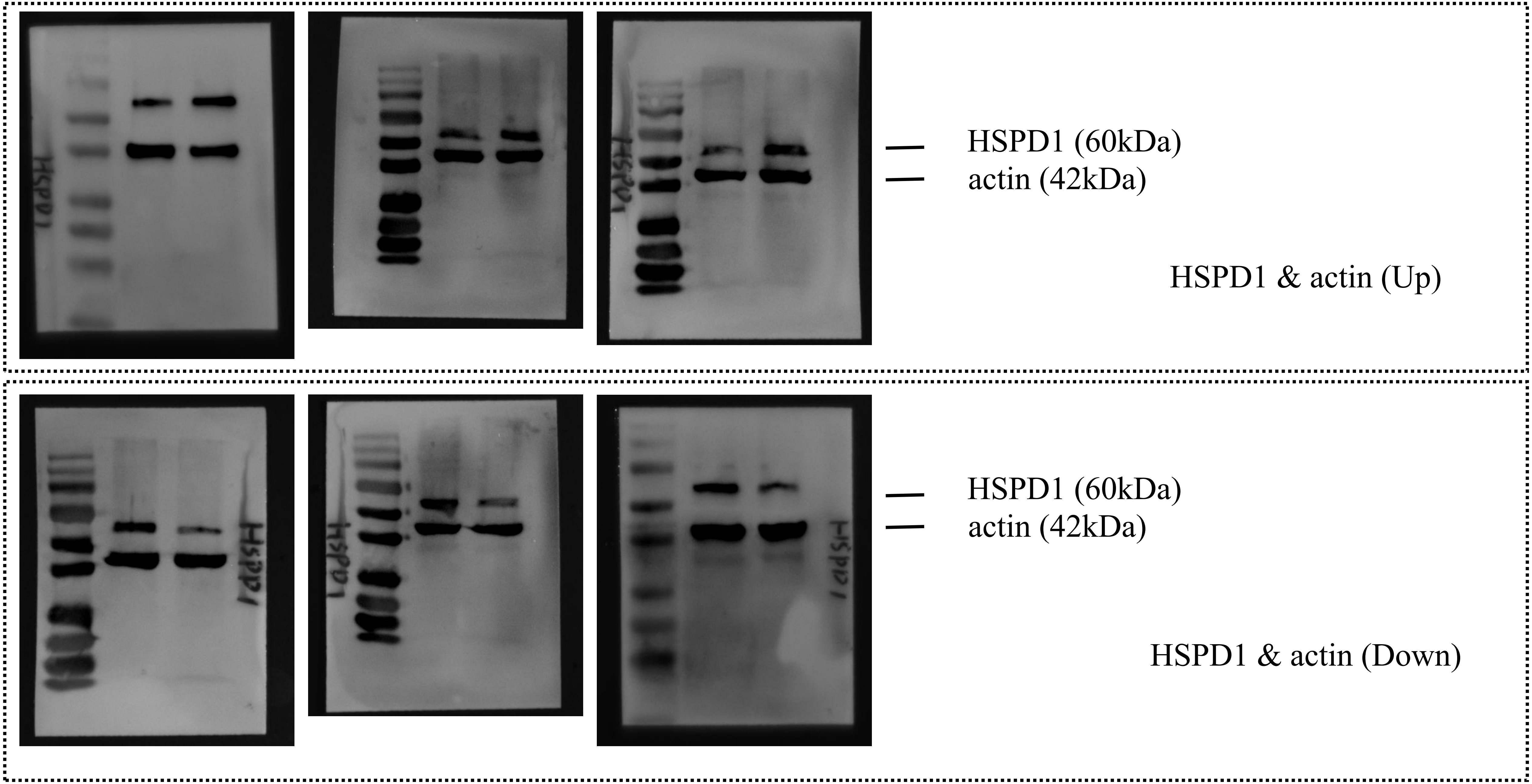

Figure S8

S7D

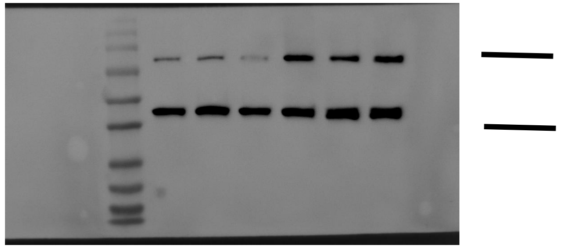

env & actin

Figure S7

7G cytoplasm

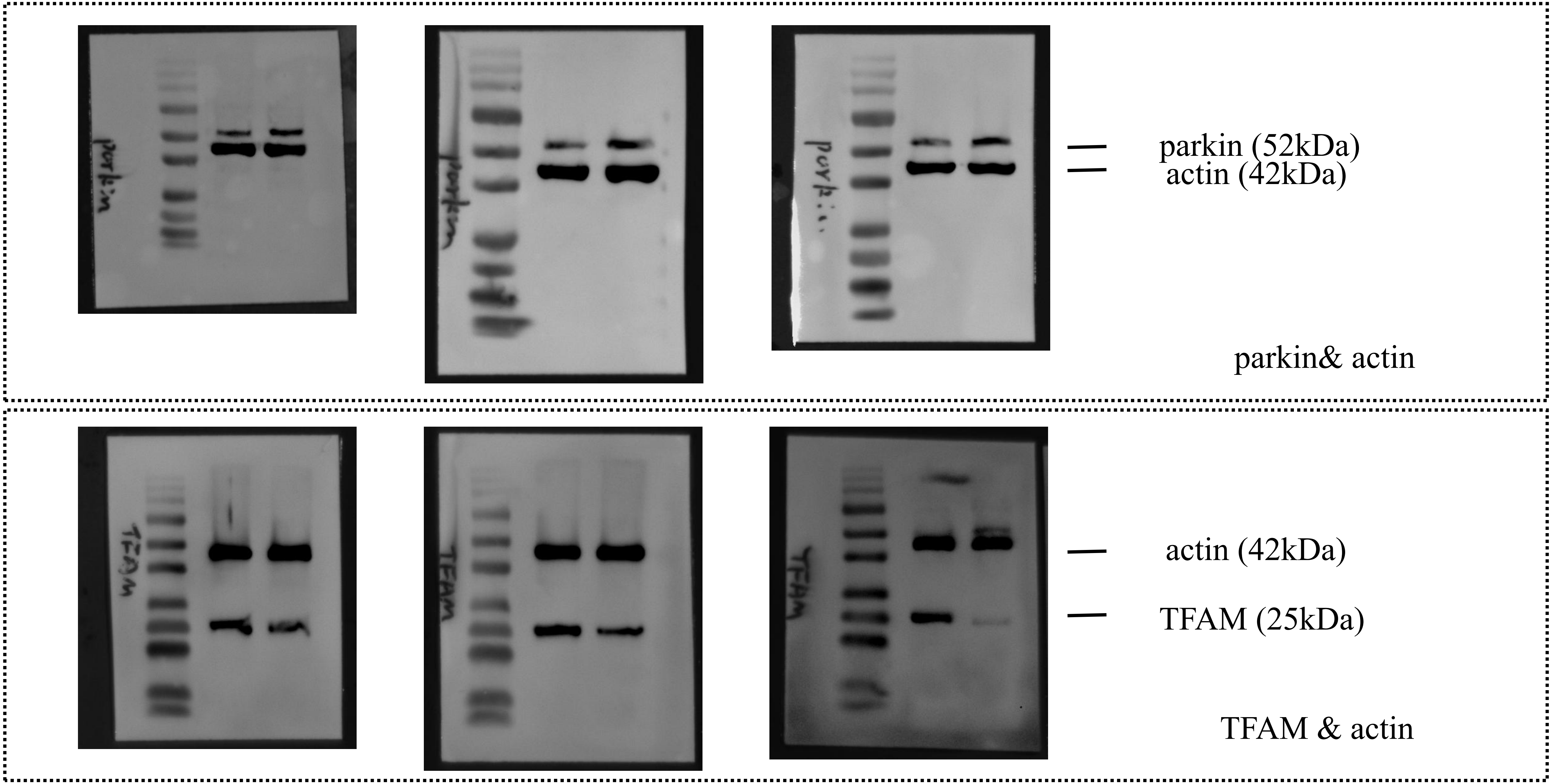

Figure S7

7G cytoplasm

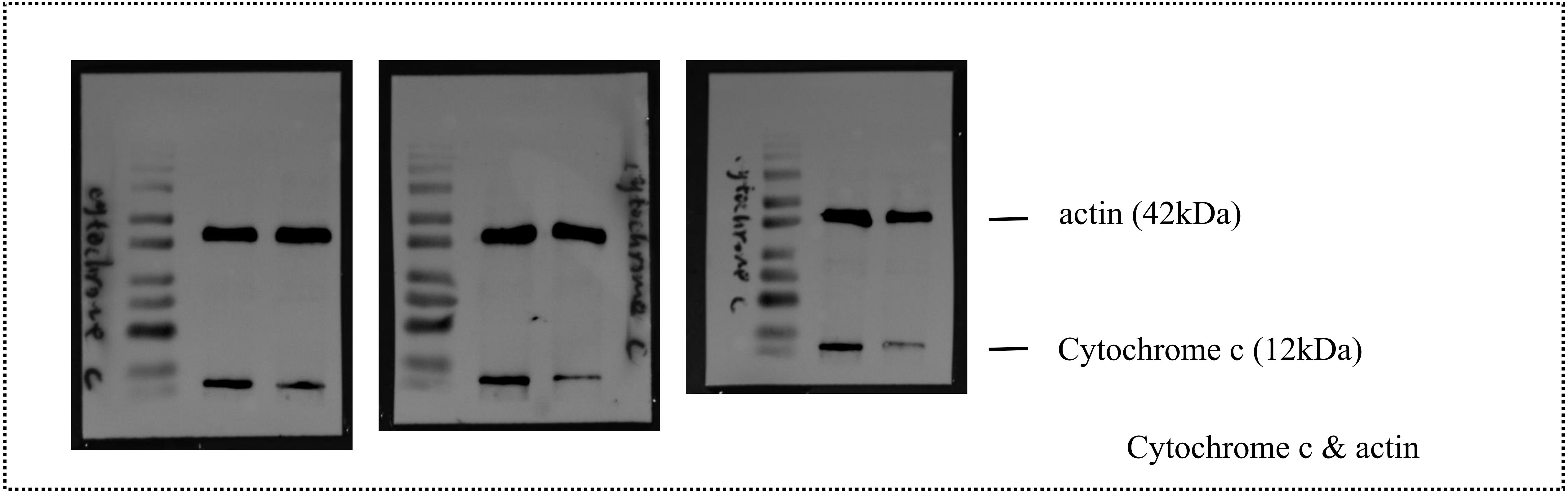

Figure S8

S8G mitochondria

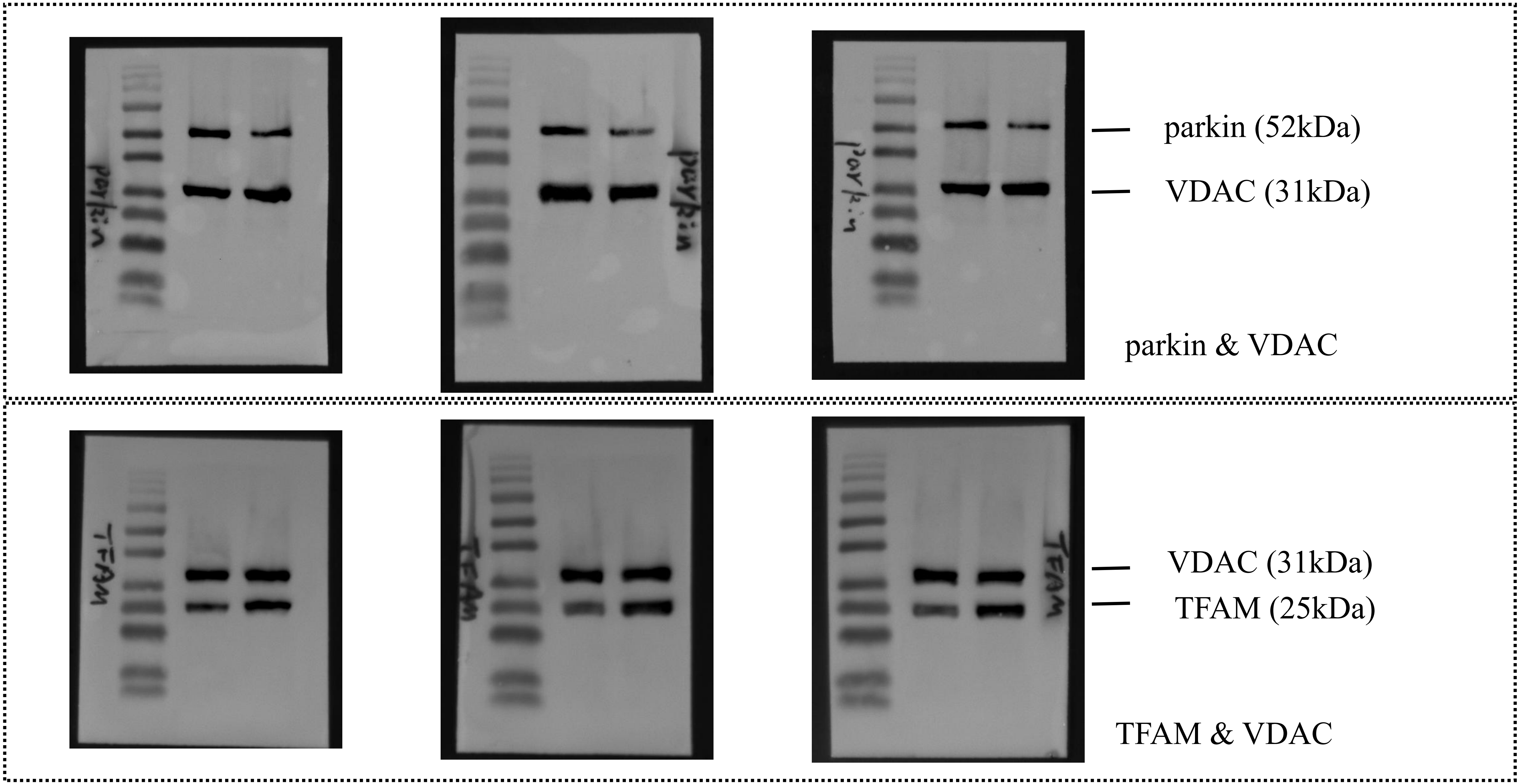

Figure S8

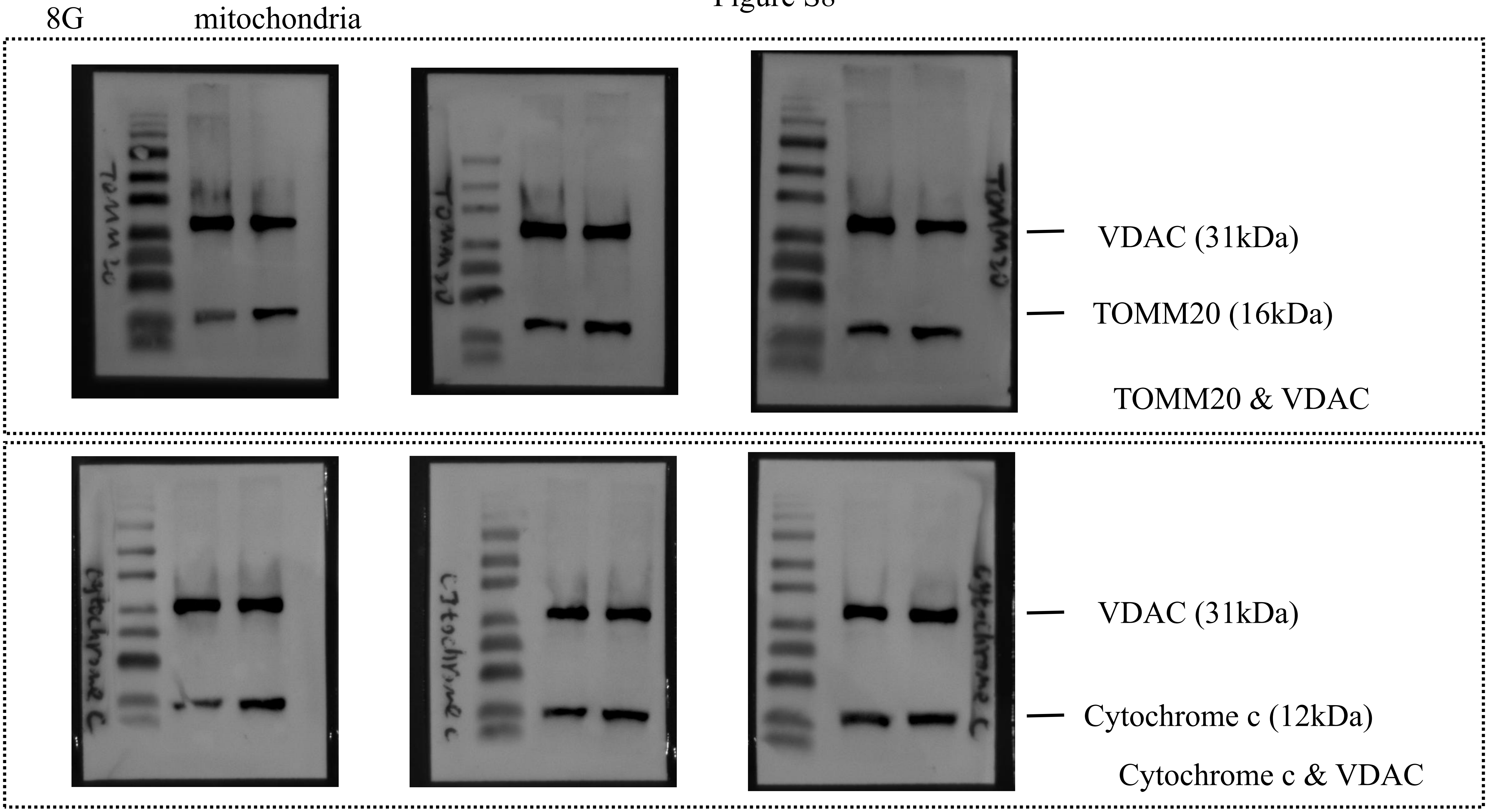
8G
mitochondria
VDAC (31kDa)
TOMM20 (16kDa)
TOMM20 & VDAC
VDAC (31kDa)
Cytochrome c (12kDa)
Cytochrome c & VDAC

Figure S7

7I

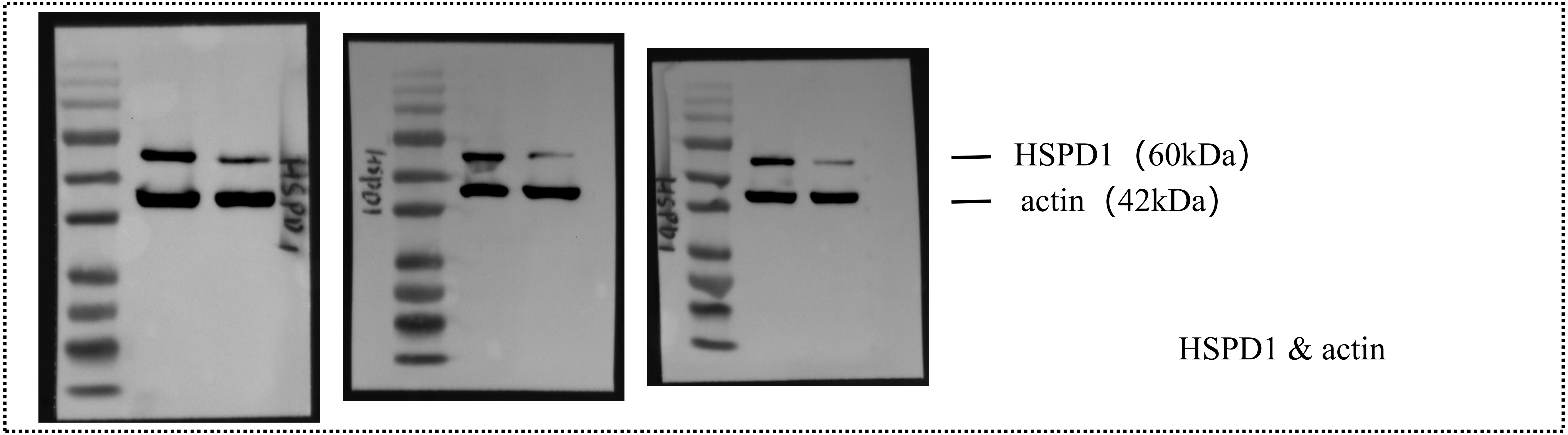
HSPD1（60kDa)
actin（42kDa)
HSPD1 & actin

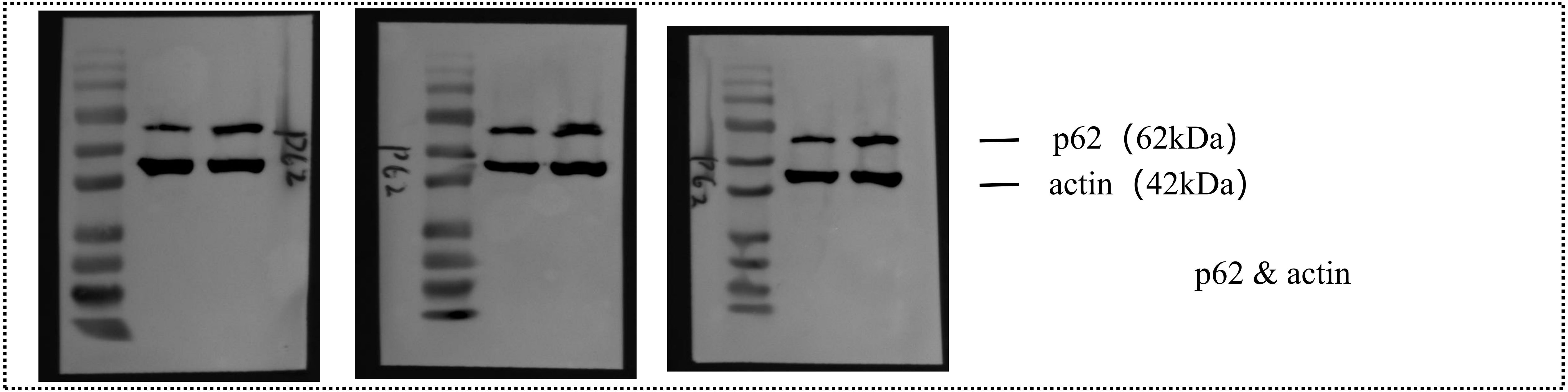
p62（62kDa)
actin（42kDa)
p62 & actin

Figure S7

S7I

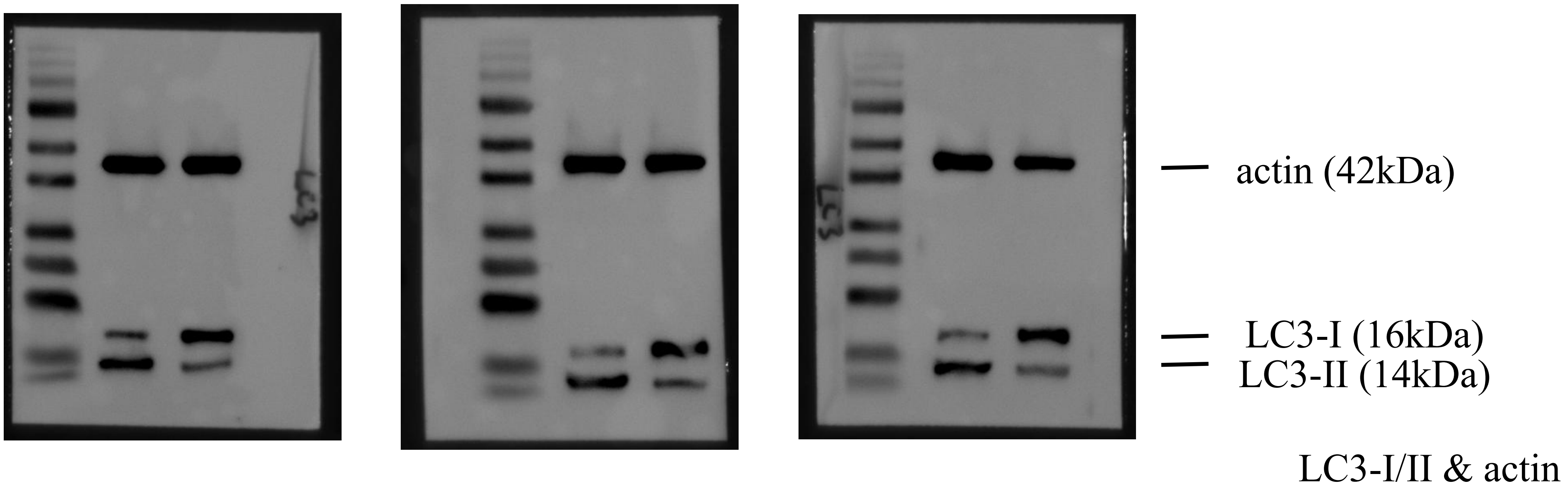

Supplement: Supplementary file 2 [file SupplementaryFile2.pdf]
